# Evaluating the effectiveness of selection indices and their genomic prediction using environmental and historical rice data

**DOI:** 10.1093/g3journal/jkaf087

**Published:** 2025-04-16

**Authors:** José Crossa, J Jesus Cerón-Rojas, Abelardo Montesinos-López, Osval A Montesinos-López, Jomar Punzalan, Adam Famoso, Roberto Fritsche-Neto

**Affiliations:** Colegio de Postgraduados, Post-grado en Estadistica y en Economia, Montecillos, Edo. de México CP 56230, México; International Maize and Wheat Improvement Center (CIMMYT), Biometrics, Quantitative Genetics and Statistics Unit, Km 45, Carretera México-Veracruz, Edo. de México CP 52640, México; International Maize and Wheat Improvement Center (CIMMYT), Biometrics, Quantitative Genetics and Statistics Unit, Km 45, Carretera México-Veracruz, Edo. de México CP 52640, México; Centro Universitario de Ciencias Exactas e Ingenierías (CUCEI), Universidad de Guadalajara, Guadalajara, Jalisco 44430, México; Facultad de Telemática, Universidad de Colima, Colima, Colima 28040, México; Louisiana State University, AgCenter 1373 Caffey Road, Rayne, LA 70578, USA; Louisiana State University, AgCenter 1373 Caffey Road, Rayne, LA 70578, USA; Louisiana State University, AgCenter 1373 Caffey Road, Rayne, LA 70578, USA

**Keywords:** genomic breeding values, genomic relationship matrix, maximum likelihood estimation, predicting unobserved index values, selection indices efficiency, genomic prediction

## Abstract

Improving genetic gains in rice breeding programs requires accurate prediction methods for selection indices. Effective use of genomic prediction could significantly accelerate breeding cycles. The Smith index method (SIM), the eigenvalue selection index method (ESIM), and the desired gain index (DG) are linear combinations of trait phenotypic values y ($I=b^{\prime }y$), and while the SIM and ESIM predict the net genetics merit ($H=w^{\prime }c$), where ***w*** is the vector of economic weights and c is the unobserved genotypic values, the DG predicts the mean of genotypic values. To enhance genomic prediction accuracy, mixed linear and Bayesian models incorporate molecular markers to estimate genomic effects, resulting in genomic estimated breeding values. This study evaluated (1) the efficiency of the SIM, ESIM, and DG through their main parameters and (2) the predictive accuracy of 5 genomic prediction models utilizing historical rice (*Oryza sativa*) data from 2018 to 2021 to predict selection indices for 2022. The correlation between observed and predicted indices assessed the effectiveness of each genomic model. Models incorporating year-specific and environmental covariates significantly improved predictive performance. These findings underscore the importance of environmental covariates and indicate that the SIM is the most effective method for maximizing key index parameters, while the ESIM provides the best predictive accuracy for indices. Consequently, rice breeders are encouraged to use these indices to enhance genetic gains per selection cycle.

## Introduction

The Smith Index Method (SIM) (Smith 1936) is an essential tool in plant and animal breeding programs. This index can be a linear combination of traits phenotypic (y) values ($I=b^{\prime }y$, where $b^{\prime }$ is a vector of optimum weights) or a linear combination of genomic estimated breeding values (Ceron-Rojas *et al*. 2015). One of the main objectives of the SIM is to predict the net genetic merit ($H=w^{\prime }c$) of an individual’s cultivar, which is a linear combination of unobservable trait additive genotypic or breeding values (c), where $w^{\prime }$ is a vector of trait economic weights. The *H* reflects an individual plant's inherent genetic potential to excel in specific desirable traits, such as yield, disease resistance, drought tolerance, or nutritional quality. A higher *H* value indicates a greater likelihood that the plant will pass on these advantageous traits to its offspring.

In the plant breeding context, Smith (1936) developed the SIM theory assuming that y and *H* have a joint multivariate normal distribution and that the vector c has multivariate normal distribution with a mean of zero and a covariance matrix C. By the first assumption, the conditional expectation of *H* given y ($E(w^{\prime }g|y)=b^{\prime }(y-\mu )=I$, where μ is the expectation of y and b is the vector of coefficients) is the Smith (1936) index.

One of the main characteristics of the SIM is that it allows extra merit in one trait to offset slight defects in another. Thus, with its use, individuals with very high merit in one trait are saved for breeding, even when they are inferior in other traits (Hazel and Lush 1942). The SIM theory has been extended to marker (Lande and Thompson 1990) and to genomic (Togashi *et al*. 2011; Ceron-Rojas *et al*. 2015) selection contexts. The main advantage of the genomic selection index over the other indices lies in the possibility of reducing the intervals between selection cycles by more than two-thirds (Cerón-Rojas and Crossa 2018).

The main SIM parameters are b, the selection response (R), the correlation between I and H ($\rho_{HI}$), and the expected genetic gain per trait (E). The selection response was defined by Smith (1936) as the mean improvement in H associated with the selected values of I. In practice, the **maximized**  R is the standard deviation of the variance of I ($\sigma_{I}$) multiplied by the selection intensity, whereas $\rho_{HI}$ is the proportion of the variance of H attributed to the regression relationship with I. In turn, E is the conditional expectation of c given I divided by the proportion of selection (Kempthorne and Nordskog 1959). All indices associated with the SIM have similar parameters but not all of them have statistical sampling properties (Cerón-Rojas and Crossa 2018, 2022).

The SIM allows breeders to jointly improve several traits that differ in additive variability, heritability, economic importance, and correlation among their phenotypes and genotypes values (Hazel *et al*. 1994). In addition, when the phenotypic covariance matrix (P) and C are known, the SIM vector of coefficients is known ($b=P^{-1}Cw$), the SIM is the best linear predictor of *H* (Cochran 1951; Henderson 1963; Cerón-Rojas and Crossa 2022), its parameters are optimums, and this index considers the multi-trait heritability ($P^{-1}C$, where $P^{-1}$ is the inverse matrix of P) when assigning trait weights; that is, the SIM vector of coefficients $b=P^{-1}Cw$ maximize the SIM and its parameters. Nevertheless, trait economic weights are difficult to assign (Bulmer 1980; Van Vleck 1993); due to this, modified indices, such as the eigen selection index method, ESIM (Cerón-Rojas *et al*. 2008, 2016) and the desired gain (DG) index (Pesek and Baker 1969; Yamada *et al*. 1975; Itoh and Yamada 1986), among others, have been proposed to solve the problem of economic weights.

The ESIM is based on the canonical correlation (Hotelling 1936), on the singular value decomposition, and on the SIM theory (Cerón-Rojas and Crossa 2018, 2022). The main ESIM characteristics are that: (1) due to the canonical correlation theory, it does not require that the economic weights are known; (2) the first eigenvector of the multi-trait heritability matrix ($P^{-1}C$) is used as its vector of coefficients, (3) due to the eigen analysis properties, it is possible to use the theory of similar matrices to change the direction and proportion of the E values without affecting the correlation between *H* and *I* (Harville 1997; Watkins 2002), and (4) in the ESIM, the selection response, correlation, and expected genetic gain per trait are obtained in a similar manner as in the SIM context (Cerón-Rojas and Crossa 2018, 2022).

The DG selection index maximizes the genetic gain for multiple traits while considering each trait's desired direction of change. This index allows breeders to specify their breeding goals and constraints explicitly, which can be advantageous for complex breeding programs targeting multiple traits. Thus, when the vector of economic weights is unknown, ESIM and the DG index might be preferable to SIM. However, Cerón-Rojas and Crossa (2018, Chapter 3) have indicated that, in the DG context, the DG vector (d) is chosen arbitrarily. Hence, we are in the same situation when economic weights need to be selected and these values are chosen arbitrarily. Pesek and Baker (1969) argued that selecting d should not be a problem for experienced breeders because they must know the relative merits and demerits of their strains. However, this may be true only for some breeders, whereas for others, the selection of d will be subjective and arbitrary.

Additionally, contrary to ESIM and SIM, the DG index does not maximize the selection response, nor its correlation with the net genetic merit because the covariance between DG and *H* is not defined. In this last case, it is only possible to estimate E where breeders impose the desired gains in each selection cycle (Pesek and Baker 1969; Itoh and Yamada 1986). Furthermore, a complete theory of this index has not been developed and the sampling properties of the estimator of E are unknown.

Before the year 2001, breeders predicted c based only on y values using the best linear unbiased predictor (BLUP) (Henderson 1963; Robinson 1991) as $E(c|y)=\hat{c}$, where E(c|y) and $\hat{c}$ denote the conditional expectation of c given y and the predicted value of c, respectively. A similar approach was used in the Bayesian context (Sorensen and Gianola 2002) where through the posterior distribution of c, breeders predicted this vector using y and the prior information of c. However, Meuwissen *et al*. (2001) showed that the incorporation of molecular makers in the mixed linear model equations to obtain BLUPs, or on the posterior distribution of c, increase the prediction accuracy. In this case, the molecular markers BLUP are denoted as GBLUP (genomic BLUP) and the predicted values ($\hat{c}$) are called genomic estimated breeding values. This selection procedure was called genomic selection by Meuwissen *et al*. (2001).

Bayesian methods have become essential in genomic prediction because they incorporate prior knowledge and model uncertainty (Meuwissen *et al*. 2001; de los Campos *et al*. 2009; Gianola *et al*. 2009; Crossa *et al*. 2010, 2011). These methods provide a probabilistic framework that allows for the estimation of complex genetic architectures by integrating genetic markers and phenotypic data, and are popular because they can effectively handle large-scale genetic markers data, accommodate different genetic architectures, and provide posterior distributions for model parameters, offering insights into the uncertainty of predictions.

A problem not considered by breeders until now is the prediction of selection index values in the genomic breeding context. This might be particularly valuable because selection index values are predictors of the net genetic merit of individuals by integrating multiple traits weighted by their economic importance and genetic correlations. When prediction methods are applied to selection indices, they effectively predict the total genetic merit of individuals, capturing the combined additive genetic contributions across the genome for all traits included in the index. This approach should enhance breeding efficiency by allowing breeders to target aggregate genetic improvement rather than individual traits, leveraging the full potential of genomic data to accelerate genetic gains (Meuwissen *et al*. 2001; Dekkers 2007). Moreover, the genomic prediction of selection index values ensures that marker effects are utilized to maximize genetic merit across traits, facilitating optimal decision-making in both plant and animal breeding programs.

Given the complexity and variability in genomic prediction modeling, there is no single, universally optimal model to predict future performance in plants. Therefore, selecting and evaluating multiple genomic prediction models based on assumptions like multivariate normality of data, genotypes, and marker effects is essential. Considering these factors, the main objectives of this research were given as follows: (1) to evaluate the efficiency of SIM and ESIM in predicting *H*, estimating *R*, $\rho_{HI}$, E (and DG in predicting E), over 4 years for 2 different sets of trait weights applied to 4 rice traits: Chalk, Whole, Ratoon, and Yield, respectively, used in the indices prediction; (2) to perform a genomic prediction of the SIM, ESIM, and DG values in the final year of the study (2022) using data from the previous 4 years (2018, 2019, 2020, and 2021), including environmental covariables for each of the years 2018–2021; (3) based on the practical results obtained in this study, the authors discussed the theoretical basis of the 3 indices and their advantages and possible disadvantages.

## Methods, models, and materials

In this work, we use 5 genomic prediction models and extensive historical rice (*Oryza sativa*) datasets from 2018 to 2021 corresponding to the conventional combination of rice grain (LONG) and conventional (HERBICIDE) practices to predict selection index values in the year 2022, and we used the correlation between the observed and predicted indices values to evaluate the efficiency of each model. The number of rice cultivars each year is different and fluctuates, on average, between 200 and 400 varieties.

## Selection indices theory

### The Smith (1936) index theory

Let $y_{i}^{\prime }=\left[\begin{array}{cccc} Y_{i1} & Y_{i2} & \cdots & Y_{it} \end{array}\right]$ and $c_{i}^{\prime }=\left[\begin{array}{cccc} C_{i1} & C_{i2} & \ldots & C_{it} \end{array}\right]$ (*i* = 1, 2, …, *n*; where *n* denotes the number of individuals or genotypes in the population) be vectors of t observable phenotypic values and their associated unobservable genotypic values, distributed as a joint multivariate normal distribution with vector of means $\mu^{\prime }=\left[\begin{array}{cccc} \mu_{1} & \mu_{2} & \ldots & \mu_{t} \end{array}\right]$ and $E(c_{i})=0$, and covariance matrix P=C+Ξ and C, respectively, where Ξ is a matrix which contains all nongenetic effect variances associated with the traits values (Hazel and Lush 1942). Note that the covariance between $y_{i}^{\prime }$ and $c_{i}^{\prime }$ is C (Smith 1936). In an environment, the relationship between $Y_{ij}$ and $C_{ij}$ (*j* = 1, 2, …, *t*; *t* is the number of traits) is


(1) $$Y_{ij}=\mu_{j}+C_{ij}+\varepsilon_{ij},$$

where $Y_{ij}$ and $C_{ij}$ denote the *j*th trait (*j* = 1, 2, …, *t*) record and its associated genotype value, respectively, on the *i*th individual, $\mu_{j}$ is the expectation of the *j*th trait, and $\varepsilon_{ij}$ denotes the residual, which has a normal distribution, null mean, and a variance of $\sigma_{\varepsilon }^{2}$. It is assumed that the covariance between $C_{ij}$ and $\varepsilon_{ij}$ is zero, that is, $\varepsilon_{ij}$ is independently distributed from the $C_{ij}$ values (Smith 1936; Hazel and Lush 1942). Note that Eq. (1) is a linear mixed model (Lynch and Walsh 1998, Chapters 26 and 27).

### The SIM and the net genetic merit

The SIM ($I_{i}$) and the net genetic merit ($H_{i}$) can be written as


(2) $$I_{i}=b^{\prime }(y_{i}-\mu )$$

and


(3) $$H_{i}=w^{\prime }c_{i},$$

respectively, where $b^{\prime }=\left[\begin{array}{cccc} b_{1} & b_{2} & \cdots & b_{t} \end{array}\right]$ denotes the vector of coefficients and $w^{\prime }=\left[\begin{array}{cccc} w_{1} & w_{2} & \ldots & w_{t} \end{array}\right]$ is the known vector of trait genotype weights or economic weights; *t* denotes the number of traits on $I_{i}$ (i=1,2,…,n; *n* is the number of individuals or genotypes), whereas $y_{i}$, μ, and $c_{i}^{\prime }$ are defined in Eq. (1). Because $y_{i}$ and $c_{i}^{\prime }$ have a joint multivariate normal distribution, Eqs. (2) and (3) have univariate normal distributions with a mean of 0 and variance $\sigma_{I}^{2}=b^{\prime }Pb$ and $\sigma_{H}^{2}=w^{\prime }Cw$, respectively, whereas the joint distribution of $I_{i}$ and $H_{i}$ is bivariate normal.

### The maximized SIM selection response, correlation, and expected genetic gain per trait

The vector of coefficients that maximized selection response (*R*), the correlation between $I_{i}$ and $H_{i}$ ($\rho_{IH}$), and expected genetic gain per trait (E) is


(4) $$b=P^{-1}Cw,$$

where C and w are defined earlier, and $P^{-1}$ is the inverse of matrix P. Equation (4) is shaped by the multi-trait heritability ($P^{-1}C$) and by w. Cerón-Rojas and Crossa (2018, 2022) give details of the derivation process to obtain Eq. (4).

By Eq. (4), the covariance between $I_{i}$ and $H_{i}$ ($\sigma_{IH}=w^{\prime }Cb$) and the variance of $I_{i}$ ($\sigma_{I}^{2}=b^{\prime }Pb$) are the same. Therefore, the selection response (*R*) can be written as


(5) $$R=\;k\sqrt{b^{\prime }Pb},$$

where *k* is the selection intensity and $\sigma_{I}=\sqrt{b^{\prime }Pb}$ is the standard deviation of the variance of $I_{i}$. In addition, the correlation between $I_{i}$ and $H_{i}$ ($\rho_{IH}$) is


(6) $$\;\rho_{HI}=\frac{\sqrt{b^{\prime }Pb}}{\sqrt{w^{\prime }Cw}}\;,$$

where all the other terms are defined earlier. In turn, the expected genetic gain per trait is


(7) $$E=k\frac{Cb}{\sqrt{b^{\prime }Pb}},$$

where all the terms of Eq. (7) are defined earlier.

### The eigen selection index method

In the ESIM, it is assumed that the selection intensity (*k*) and the net genetic merit (Eq. 3) variance ($\sigma_{H}^{2}$) are fixed, whereas matrices C and P are known. To obtain the ESIM vector of coefficients that maximizes the selection response (Eq. 5), correlation (Eq. 6), and expected genetic per trait (Eq. 7), it is necessary to maximize the square correlation between Eqs. (2) and (3), i.e.


$$\rho_{HI}^{2}=\frac{{\left(w^{\prime }Cb\right)}^{2}}{\left(w^{\prime }Cw)(b^{\prime }Pb\right)}$$

with respect to vectors b and w under the restrictions $\sigma_{I}^{2}=b^{\prime }Pb$, $\sigma_{H}^{2}=w^{\prime }Cw$ and 0 < $\sigma_{H}^{2}$, $\sigma_{I}^{2}$ <∞, where $\sigma_{I}^{2}=b^{\prime }Pb$ is the variance of Eq. (2) and $\sigma_{H}^{2}=w^{\prime }Cw$ is the variance of Eq. (3).

Cerón-Rojas and Crossa (2018, Chapter 7) showed that the vector ($b_{E}$) that maximizes the ESIM selection response, correlation with Eq. (3) and expected genetic gain per trait (Eq. 7) can be obtained from the following equation:


(8) $$(P^{-1}C-\lambda_{E}^{2}I)b_{E}=0$$

or from the equation:


(9) $$(T-\lambda_{E}^{2}I)\beta =0,$$

where $T=FP^{-1}CF^{-1}$, $\beta =Fb_{E}$, and $F=diag\left\{\begin{array}{cccc} \;f_{1} & \;f_{1} & \cdots & \;f_{t} \end{array}\right\}$ is a diagonal matrix with values equal to any real number, except values of zero. Matrix $T=FP^{-1}CF^{-1}$ is called the *similarity transformation matrix*, whereas matrix F is called the *transforming matrix* (Watkins 2002). Harville (1997) indicated that T and $P^{-1}C$ are similar matrices and that both have the same eigenvalues, although different eigenvectors. When the F values are only 1's, vector $\beta =Fb_{E}$ is not affected by matrix F and $\beta =b_{E}$ (Eq. 8), yet if the F values are only negatives (−1's), vector $\beta =Fb_{E}$ (Eq. 9) will change its direction. In addition, if the F values are different to 1 and −1, matrix F will change the proportional values of $\beta =Fb_{E}$. In practice, $b_{E}$ is first obtained from Eq. (8) and then multiplied by matrix F to obtain $\beta =Fb_{E}$ (Eq. 9), that is, β is a linear transformation of $b_{E}$.

The ESIM selection response, correlation, and E are obtained in a similar manner as in the SIM context changing vector b by vector $\beta =Fb_{E}$ in Eqs. (5)–(7), whereas the ESIM index is $I_{E}=\beta^{\prime }y$; Eq. (3) is $H_{E_{i}}=w_{E}^{\prime }c_{i}$, and $\mathrm{var}(H_{E})=w_{E}^{\prime }Cw_{E}$, where $w_{E}=C^{-1}P\beta$ (Cerón-Rojas and Crossa 2018, Chapter 7).

### DG selection index

This index is based on Eq. (7) and its most important aspect is that it does not require economic weights (Itoh and Yamada 1986). Pesek and Baker (1969) indicated that if Cb is to be written as


Cb=d

where d is a vector of desired gains imposed by the breeders, then Eq. (7) can be written as


(10) $$E=k\frac{d}{\sigma_{I}},$$

where all the terms are defined earlier. According to Brascamp (1984) and Itoh and Yamada (1986), Eq. (10) is inversely proportional to the standard deviation of the variance of the index ($\sigma_{I}$). Hence, if we minimize $\sigma_{I}$ with respect to b subject to the constraints Cb=d, we should maximize E. Thus, assuming that P, C, and d are known, we can take the derivative of the function


$$\Phi (b,v)=0.5(b^{\prime }Pb)+v^{\prime }(Cb-d)$$

with respect to b and v, where v is a vector of Lagrange multipliers. Itoh and Yamada (1986) showed that the vector that minimizes $\sigma_{I}$ and maximizes E is


(11) $$b_{DG}=P^{-1}C(CP^{-1}C{)}^{-1}d.$$

In Eq. (11), it is assumed that the traits in the index are the same as those in the net genetic merit. Note that because Eq. (11) minimizes $\sigma_{I}^{2}=b^{\prime }Pb$, the estimated values of $\sigma_{I}^{2}$ might be negatives, and in such case, Eq. (10) will not have any real value.

## The vector of the individual genomic breeding values and the genomic relationship matrix

The vector of the individual genomic breeding values ($g_{j}$) associated with the *j*th characteristic (j=1, 2, …, *t*; *t* is the number of traits) of the candidates for selection can be written as


$$g_{j}=Xu_{j}$$

where X is an n×m matrix (*n* is the number of observations and *m* is the number of markers in the population) of coded marker values (2−2p, 1−2p, and −2p for genotypes *AA*, *Aa*, and *aa*, respectively, where *p* is the frequency of allele *A* and 1−p is the frequency of allele *a*) associated with the additive effects of the quantitative trait loci (QTL) and $u_{j}$ is an m×1 vector of the additive effects of the QTL associated with markers that affect the *j*th trait. It is assumed that $g_{j}$ has multivariate normal distribution (MVN) with a mean of **0** and a variance of $G\sigma_{g_{j}}^{2}$, i.e. $g_{j}$ ∼ MVN (**0**, $G\sigma_{g_{j}}^{2}$), where $\sigma_{g_{j}}^{2}$ is the additive genomic variance of $g_{j}$ and $G={XX}^{\prime }/\pi$ is the n×n additive genomic relationship matrix between individuals; $\pi =\overset{m}{\underset{q=1}{\sum }}\;2p_{q}(1-p_{q})$ in an F_2_ population, and $\pi =\overset{m}{\underset{q=1}{\sum }}\;4p_{q}(1-p_{q})$ in a double haploid population.

For *t* traits, the vector of the individual genomic breeding values ($g_{j}$) can be written as $g^{\prime }=\left[\begin{array}{cccc} g_{1}^{\prime } & g_{2}^{\prime } & \ldots & g_{t}^{\prime } \end{array}\right]$, or assuming that $G_{j}$ (j=1, 2, …, *t*) is a random variable which can take any value of $g_{j}=Xu_{j}$, we might have the random vector $g^{\prime }=\left[\begin{array}{cccc} G_{1} & G_{2} & \ldots & G_{t} \end{array}\right]$. In addition, because $g_{j}=Xu_{j}$ is associated to the *j*th trait, Eq. (2) can be written as $I_{i}=b^{\prime }(y_{i}-\mu )\approx b^{\prime }g_{i}$, where $g_{i}^{\prime }=\left[\begin{array}{cccc} G_{i1} & G_{i2} & \ldots & G_{it} \end{array}\right]$ (i=1,2,…,n; *n* is the number of individuals or genotypes) and ≈ denotes an approximation to Eq. (2). In this case, the variance of $I_{i}\approx b^{\prime }g_{i}$ can be written as $\mathrm{var}(I_{i})\approx \mathrm{var}(b^{\prime }g_{i})=b^{\prime }\Gamma b$, where $\Gamma \approx \mathrm{var}(g_{i})=\{\sigma_{\;jh}\}$ is a matrix of size t×t and $\sigma_{\;jh}$ is the additive genomic covariance of $g_{j}$ and $g_{h}$ (j,h=1, 2, …, *t*) (Cerón-Rojas and Crossa 2018, p. 101). It is assumed that the covariance between $g_{ij}$ and $c_{ij}$ (Eq. 1) is $\mathrm{cov}(g_{ij},c_{ij})=\sigma_{g_{j}}^{2}$ (Dekkers 2007).

## The 5 models to predict selection indices values

To evaluate the performance of the SIM, ESIM, and DG to predict their values in the year 2022, we explored 5 genomic prediction models resulting from different considerations regarding the inclusion of available information through matrix G, years, and environment covariates, which helped control the variability in the prediction. As we shall see, each of the 5 prediction models was divided into several sub-models according to the information included in the prediction. The sub-models differ based on the years used for training (Table 1, second column).

**Table 1.** Models to predict the unobservable index values in 2022 based on genomic relationship matrix (G) and matrix on environmental covariates, using the covariate information and the available training years in different ways.

Model	Training set year	Sub-model number	Environment covariate (EC)
1	2018–2021	1	NA
1	2018 or 2019 or 2020 or 2021	2, 3, 4, 5	NA
1	2019–2021	6	NA
1	2020–2021	7	NA
2	2018–2021	8	Average of EC in training years
2	2018 or 2019 or 2020 or 2021	9, 10, 11, 12	Of training year
2	2019–2021	13	Average of EC in training years
2	2020–2021	14	Average of EC in training years
2	2018–2021	15	Of previous years
2	2019–2021	16	Of previous years
2	2020–2021	17	Of previous years
2	2018–2021	18	Of two years ago
2	2019–2021	19	Of two years ago
2	2020–2021	20	Of two years ago
3	2018–2021	21	NA
3	2019–2021	22	NA
3	2020–2021	23	NA
4	2018–2021	24	Average of training years
4	2019–2021	25	Average of training years
4	2020–2021	26	Average of training years
4	2018–2021	27	Of previous year
4	2019–2021	28	Of previous year
4	2020–2021	29	Of previous year
4	2018–2021	30	Of two years ago
4	2019–2021	31	Of two years ago
4	2020–2021	32	Of two years ago
5	2018–2021	33	Average of training years
5	2019–2021	34	Average of training years
5	2020–2021	35	Average of training years
5	2018–2021	36	Of previous year
5	2019–2021	37	Of previous year
5	2020–2021	38	Of previous year
5	2019–2021	39	Of two years ago
5	2018–2021	40	Of two years ago
5	2020–2021	41	Of two years ago

The first column lists the model, defined by the predictors including the effects considered. The second column specifies the different years used to train the base model, resulting in various sub-models (third column). The last column explains how the environmental covariates are incorporated in each sub-model. “NA” indicates that this information on EC is not included in the corresponding sub-model.

### Model 1

This model has 7 sub-models (1–7) (Table 1), which include similar information in the prediction. The absence of covariate information in this first block of models (1–7) is indicated in the last column (EC = NA) of Table 1. The ECs were obtained and processed using the envRtype R-package (Costa-Neto *et al*. 2021). Also, weather data was obtained daily, and at the end, a Gaussian kernel was built to describe the relationship between environments.

By Eq. (2), Model 1 can be written as


$$I_{ir}=\mu +s_{ir}+e_{ir}$$

where $\;I_{ir}$ is the *i*th (*i* = 1, 2, …, *n*, *r* = 1, 2, 3, i.e. SIM, ESIM, or DG) selection indices to be predicted and *μ* is the expectation of $I_{ir}$. For SIM, ESIM, and DG, $s_{ir}=\overset{t}{\underset{\;j=1}{\sum }}\;b_{j}g_{j}$, $s_{ir}=\overset{t}{\underset{\;j=1}{\sum }}\;\beta_{j}g_{j}$, and $s_{ir}=\overset{t}{\underset{\;j=1}{\sum }}\;b_{DG_{j}}g_{j}$ respectively, which have a normal distribution, a null mean, and a variance of $\sigma_{s}^{2}=b^{\prime }\Gamma b$ for SIM, $\sigma_{s}^{2}=\beta^{\prime }\Gamma \beta$ for ESIM, and $\sigma_{s}^{2}=b_{\mathrm{DG}}^{\prime }\Gamma b_{\mathrm{DG}}$ for DG. In addition, $e_{ir}$ denotes the residuals with a normal distribution, a null mean, and a variance of $I_{n}\sigma_{e}^{2}$, where $I_{n}$ is an identity matrix sized *n*, and *n* is the number of individuals or lines in the population. It is assumed that the covariance between $s_{ir}$ and $e_{ir}\;$ is zero, that is, $s_{ir}$ is independently distributed from the $e_{ir}$ values. This means that the variance of Model 1 should be $V_{I_{r}}=G\sigma_{s}^{2}+I_{n}\sigma_{e}^{2}$.

### Model 2

The next 13 sub-models (8–20) (Table 1) not only include $s_{ir}$ (Model 1) but also incorporate the environmental covariates through the random effects $g_{YCovs_{ir}}$. This model can be written as


$$I_{ir}=\mu +s_{ir}+g_{YCovs_{ir}}+e_{ir},$$

where, once again, $I_{ir}$ is the *i*th selection indices to be predicted and *μ* is the expectation of $I_{ir}$. It is assumed that $g_{YCovs_{ir}}$ (*i* = 1, 2, …, *n*; *r* = 1, 2, 3, i.e. SIM, ESIM, or DG) is jointly distributed as a multivariate normal with a vector of null means and variance $\sigma_{YC}^{2}\;K_{LYC}$, that is, the vector of random effects $g_{YCovs_{ir}}\;∼\;N(0,\;\sigma_{YC}^{2}\;K_{LYC})$, where “∼” *denotes distributed as*, and $K_{LYC}$ is the linear kernel used to measure the similarity between years based on the year environmental covariables $H=\frac{{SS}^{\prime }}{N_{\mathrm{bands}}}$, where S is a matrix of the centered and standardized mean for the hyperspectral bands, and $N_{\mathrm{bands}}$ is the total number of hyperspectral bands. Again, $s_{ir}$ and $e_{ir}$ are random effects as defined in Model 1. In a similar manner to Model 1, we assumed that the covariance between the terms $s_{ir}$, $g_{YCovs_{ir}}$, and $e_{ir}$ is zero, that is, they are independently distributed by pairs, from where the variance of Model 2 is $V_{I_{r}}=G\sigma_{s}^{2}+\sigma_{YC}^{2}K_{LYC}+I_{n}\sigma_{e}^{2}$.

Model 2 sub-models (Table 1) also differ from each other based on the years used for training, as specified in the second column of Table 1 (Training set), and how the values of the environmental covariates for the target year are handled. For example, sub-model 8 includes the effect of lines ($s_{ir}$) and the effect of environmental variables ($g_{YCovs_{ir}}$), and is trained with all previous years (2018–2021). The environmental covariate (EC) values used for 2022 are the average of the environmental covariates from all the training years. In this case, EC = Average of EC in training years.

Sub-models 9–12 are trained for only 1 year, and the value of the EC for the target year is the same as that of the training year (EC = Of training year). The cells with the value “*Of previous years*” indicate that the EC values for the target year are taken to be the same as those of the prior year (2021), while if EC = *Of two years ago*, the corresponding EC values for the target year (2022) are taken to be the same as those from the year 2020.

### Model 3

The next 3 sub-models (sub-models 21–23) (Table 1) are extensions of sub-models 1, 6, and 7, respectively, where the year is now considered as a trend effect ($\beta_{Y}Year_{ir}$). This model can be written as


$$I_{ir}=\mu +\beta_{Y}Year_{ir}+s_{ir}+e_{ir},$$

where, once again, $I_{ir}$ is the *i*th selection indices to be predicted and *μ* is the expectation of $I_{ir}$. The covariance between the terms $Year_{ir}$, $s_{ir}$, and $e_{ir}\;$ is zero, this means that they are independently distributed by pairs. Once again, $s_{ir}$ and $e_{ir}$ are random effects as defined in Model 1, and the variance of Model 3 is $V_{I_{r}}=G\sigma_{s}^{2}+I_{n}\sigma_{e}^{2}$.

### Model 4

Sub-models 24–32 (Table 1) are extensions of sub-models 21–23, incorporating the environmental covariates ($g_{YCovs_{ir}}$) similarly to sub-models 8–20. This model is


$$I_{ir}=\mu +\beta_{Y}Year_{ir}+s_{ir}+g_{YCovs_{ir}}+e_{ir},$$

where, again, $I_{ir}$ is the *i*th selection indices to be predicted and *μ* is the expectation of $I_{ir}$. The covariance between the terms $Year_{ir}$, $s_{ir}$, $g_{YCovs_{ir}}$, and $e_{ir}$ is zero; this means that they are independently distributed by pairs. In a similar manner as in Model 2, $g_{YCovs_{ir}}$ (*i* = 1, 2, …, *n*; *r* = 1, 2, 3, i.e. SIM, ESIM, or DG) is jointly distributed as a multivariate normal with a vector of null means and a variance of $\sigma_{YC}^{2}\;K_{LYC}$, that is, the vector of values $g_{YCovs_{ir}}\;∼\;N(0,\;\sigma_{YC}^{2}\;K_{LYC})$, where “∼” *denotes distributed as*, and $K_{LYC}$ is the linear kernel used to measure the similarity between years based on the year environmental covariables. Again, $s_{ir}$, $g_{YCovs_{ir}}$, and $e_{ir}$ are random effects as defined in Model 2, from where the variance of Model 4 is $V_{I_{r}}=G\sigma_{s}^{2}+\sigma_{YC}^{2}K_{LYC}+I_{n}\sigma_{e}^{2}$.

### Model 5

The last explored sub-models (33–41) are the same as sub-models 24–32, but now the vector of values of random effects of the environmental covariates $g_{YCovs_{ir}}\;∼\;N(0,\;\sigma_{YC}^{2}\;K_{YG})$, where $K_{YG}$ is the nonlinear Gaussian kernel used to measure the similarity of the EC values between years.

### Materials

We used 4-year (2018, 2019, 2020, and 2021) real rice datasets with a different number of rice cultivars and traits in each year (see Tables 3–5). The traits used to construct SIM, ESIM, and DG in each year were Chalk (%), Whole milling (%), Ratoon yield (ton/h), and Yield grain (ton/h). The materials come from the elite × elite breeding program for the Conventional Pipeline. For the first 2 years, it was an randomized complete block design with 2 replicates. Then, it moved to an augmented design with 2 replicates over time (spaced by a month). All trials were planted in just 1 location, given its size. Also, in all of them, the spatial analysis complemented the experimental design. Every year, about 1,200 new lines are evaluated at the F_5_ stage under field conditions, genotyped, and included in the training set dataset. Therefore, it is a brand new set of individuals, with a small overlap over the years (just a few checks).

We evaluated and compared the SIM, ESIM, DG, efficiency using the expected genetic gain per trait (**E**), the selection response (*R*), and the correlation between each index and the net genetic merit ($\rho_{HI}$). In addition, we predict the SIM, ESIM, and DG values for the year 2022 using the estimated values of the SIM, ESIM, and DG for the years 2018, 2019, 2020, and 2021, the 5 models described earlier, and the genomics breeding values ($g_{j}=Xu_{j}$) and the genomic relationship matrix (G). The estimated correlations between estimated indices and values of predicted indices were used to compare the efficiency of the 5 models (Models 1–5) of index predictions. Note that all the rice cultivars in 1 year are different to those in another year.

The selection objective was to decrease the expected genetic gain for trait Chalk, and for this reason, for the SIM, we used 2 vectors of trait weights (***w***): $w^{\prime }=\left[\begin{array}{cccc} -1 & 1 & 1 & 1 \end{array}\right]$ and $w^{\prime }=\left[\begin{array}{cccc} -10 & 10 & 10 & 70 \end{array}\right]$. In addition, for ESIM, the diagonal transforming matrix (F) used to change the proportions of the vector of coefficients (Harville 1997; Watkins 2002; Cerón-Rojas and Crossa 2018, Chapter 7) and the expected genetic gain per trait values were $F=diag\left\{-\begin{array}{cccc} 1 & 1 & 1 & 1 \end{array}\right\}$ and $F=diag\left\{\begin{array}{cccc} -10 & 10 & 10 & 70 \end{array}\right\}$, where *diag* denotes the diagonal matrix. Similarly, for the DG index, the vector of desired gains (***d***) were $d^{\prime }=\left[\begin{array}{cccc} -1 & 1 & 1 & 1 \end{array}\right]$ and $d^{\prime }=\left[\begin{array}{cccc} -10 & 10 & 10 & 70 \end{array}\right]$.

As the starting point for weights, we used the economic importance the breeder has used to perform the selections. In this case, we consider the proportion that each trait impacts the financial value of the variety to be released in the market. Thus, in this work, the sets of trait weights were assigned according to the desired expected genetic gains per trait. Ceron-Rojas *et al*. (2023) described the case in which the trait weights are assigned accordingly to the selection response advance using a profit function.

We estimated the phenotypic (P) and genotypic (C) covariance matrices using REML (restricted maximum likelihood), as described by Vattikuti *et al*. (2012), i.e. using matrix G in the place of the numerical relationship matrix to incorporate the relationship between individuals in the estimation process. In all years, matrix G was constructed with 435 molecular markers and a different number of individuals (see the last column of Tables 3 and 4). Before using the LSU 435 single nucleotide polymorphism (SNP) set in this study and in our routine genomic selection each year in our breeding program, we already underwent an optimization procedure from the 7,000 SNP set, as detailed in Cerioli *et al*. (2022). We have established that the 435 SNP set provides sufficient accuracy without compromising the effectiveness of genomic selection. There is no benefit in using the LSU 1,200 SNP set or a larger set. Breeder can use the 4.000 SNP set to get a better accuracy but the cost will be so high that it does not warrant to use in exchange for phenotyping or establishing a trial.

The estimated covariance matrices ($\hat{P}$ and $\hat{C}$) between traits Chalk and Yield grain within the year 2020 were, respectively:


$$\hat{P}=\left[\begin{array}{cc} 6.67 & 0.54\\ 0.54 & 0.71 \end{array}\right]\;\mathrm{and}\;\hat{C}=\left[\begin{array}{cc} 2.93 & 0.31\\ 0.31 & 0.36 \end{array}\right].$$

Because we have phenotypic and maker information to estimate the genotypic variance and covariance (Vattikuti *et al*. 2012), the estimated matrix $\hat{C}$ is the same as the estimated matrix $\hat{\Gamma }$. Ceron-Rojas *et al* (2015) have given details of how to estimate Γ when there is only marker information. Matrices $\hat{P}$ and $\hat{C}$ will be used to illustrate the estimation process of the parameters of the index, e.g. vector of coefficients, selection response, correlation, and expected genetic gain per trait.

## Description of figures

**Fig. 1.**
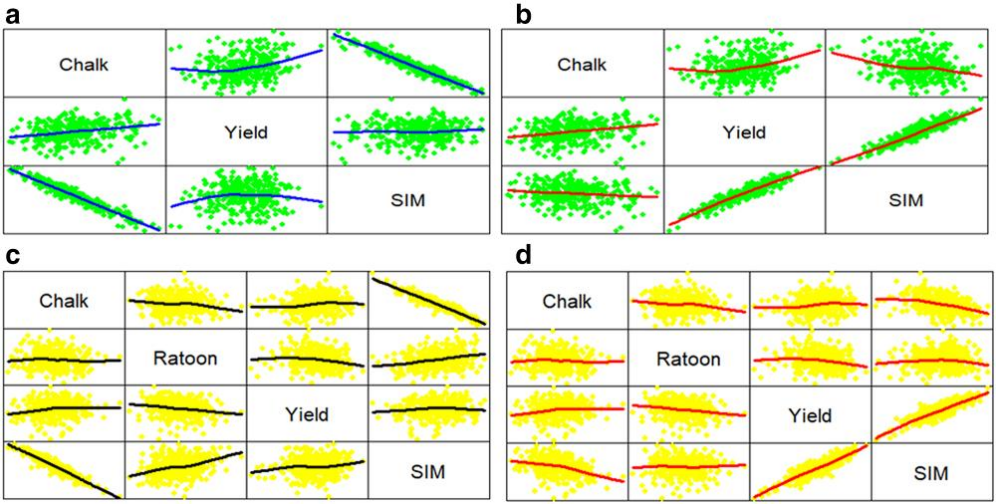
Relationship among every possible pair of traits Whole, Yield, and SIM (the year 2020) and among traits Whole, Ratoon, Yield, and SIM (the year 2021). For the year 2020, figures a and b are associated with weights $w^{\prime }=\left[\begin{array}{cc} -1 & 1 \end{array}\right]$ (a) and $w^{\prime }=\left[\begin{array}{cc} -10 & 70 \end{array}\right]$ (b), respectively, whereas for the year 2021, figures c and d are associated with weights $w^{\prime }$ = [−1  1  1] (c) and $w^{\prime }$ = [−10  10  70] (d), respectively. Data points are plotted twice, with axes reversed above and below the diagonal. A smoothed regression line, known as a loess curve, is included to highlight linear and nonlinear relationships clearly.

**Fig. 2.**
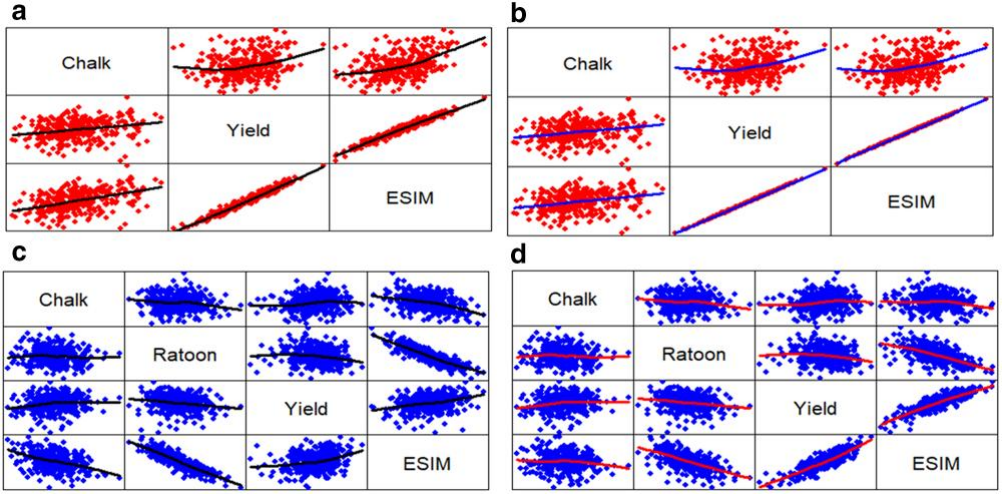
Relationship among every possible pair of traits Whole, Yield, and ESIM (the year 2020) and among traits Whole, Ratoon, Yield, and ESIM (the year 2021). For the year 2020, figures a and b are associated with weights $F=diag\left\{\begin{array}{cc} -1 & 1 \end{array}\right\}$ (a) and $F=diag\left\{\begin{array}{cc} -10 & 70 \end{array}\right\}$ (b), respectively, whereas for the year 2021, figures c and d are associated with weights ***F*** = *diag*{−1  1  1} (c) and ***F*** = *diag*{−10  10  70} (d), respectively. Data points are plotted twice, with axes reversed above and below the diagonal. A smoothed regression line, known as a loess curve, is included to highlight linear and nonlinear relationships clearly.

**Fig. 3.**
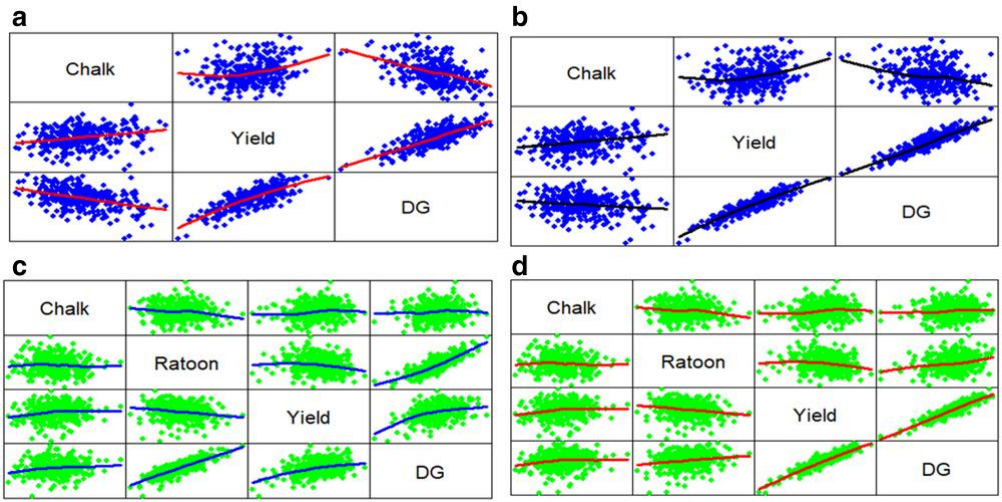
Relationship among every possible pair of traits Whole, Yield, and DG (the year 2020) and among traits Whole, Ratoon, Yield, and DG (the year 2021). For the year 2020, figures a and b are associated with weights $d^{\prime }=\left[\begin{array}{cc} -1 & 1 \end{array}\right]$ (a) and $d^{\prime }=\left[\begin{array}{cc} -10 & 70 \end{array}\right]$ (b), respectively, whereas for the year 2021, figures c and d are associated with weights $d^{\prime }$ = [−1  1  1] (c) and $d^{\prime }$ = [−10  10  70] (d), respectively. Data points are plotted twice, with axes reversed above and below the diagonal. A smoothed regression line, known as a loess curve, is included to highlight linear and nonlinear relationships clearly.

Figures 1–3 were produced using the R pairs() command (Zelterman 2015, p. 76). These figures illustrate how trait weights influence relationships between traits and selection indices. Data points are plotted twice, with axes reversed above and below the diagonal.

Supplementary Figures 1a, 2a, and 3a (Supplementary Material A) were obtained using the **corrplot.mixed()** function, which is part of the **corrplot R-package** (Wei *et al*. 2024). The link: https://cran.r-project.org/web/packages/corrplot/vignettes/corrplot-intro.html includes an introduction to the **corrplot R-package**.

## Results

### Indices estimation procedures

To illustrate the estimation process, we will obtain the estimated SIM parameters using $w^{\prime }=\left[\begin{array}{cc} -1 & 1 \end{array}\right]$. In addition, for ESIM, the diagonal transforming matrix was $F=diag\left\{\begin{array}{cc} -1 & 1 \end{array}\right\}$ and, for DG, the vector of desired gains was $d^{\prime }=\left[\begin{array}{cc} -1 & 1 \end{array}\right]$. Furthermore, we used a proportion of 10% (*k* = 1.755) to estimate the selection response and expected genetic gain per trait.

#### Estimated SIM index parameters

The estimated SIM index vector of coefficients was


$${\hat{b}}^{\prime }=\left[\begin{array}{cc} -0.42 & 0.40 \end{array}\right],$$

from which the estimated SIM for the *i*th individual was


$${\hat{I}}_{i}=-0.42(Chalk-\bar{Chalk})+0.4(Yield-\bar{Yield}),$$

where $\bar{Chalk}$ and $\bar{Yield}$ denote the average of traits *Chalk* and *Yield*, respectively.

The estimated selection response, correlation, and expected genetic gain per trait were

$\hat{R}=\;(1.755)\sqrt{\hat{b^{\prime }}\hat{P}\hat{b}}=1.97$ , $\;{\hat{\rho }}_{HI}=\frac{\sqrt{\hat{b^{\prime }}\hat{P}\hat{b}}}{\sqrt{w^{\prime }\hat{C}w}}=0.65$, and ${\hat{E}}^{\prime }=(1.755)\frac{{\hat{b}}^{\prime }\hat{C}}{\sqrt{\hat{b^{\prime }}\hat{P}\hat{b}}}=\left[\begin{array}{cc} -1.85 & 0.02 \end{array}\right]$, respectively .

#### Estimated ESIM parameters

The estimated ESIM first eigenvector of coefficients was


$$\hat{\beta }=\left[\begin{array}{cc} 0.07 & 1.0 \end{array}\right]$$

and the estimated ESIM for the *i*th individual was


$${\hat{I}}_{E_{i}}=0.07(Chalk-\bar{Chalk})+1.0(Yield-\bar{Yield}),$$

where $\bar{Chalk}$ and $\bar{Yield}$ denote the average of trait *Chalk* and *Yield*, respectively.

The estimated ESIM selection response, correlation, and expected genetic gain per trait (Cerón-Rojas and Crossa 2018, Chapter 7 for details) were


$$\begin{array}{rl} & {\hat{R}}_{E}=\;\left(1.755\right)\sqrt{{\hat{\beta }}^{\prime }\hat{P}\hat{\beta }}=1.58,\;\;{\hat{\rho }}_{H_{E}I_{E}}=\frac{\sqrt{{\hat{\beta }}^{\prime }\hat{P}\hat{\beta }}}{\sqrt{w_{E}^{\prime }\hat{G}w_{E}}}\;=0.72,\;\mathrm{and}\\ & {\hat{E}}_{E}^{\prime }=\left(1.755\right)\frac{{\hat{\beta }}^{\prime }\hat{C}}{\sqrt{{\hat{\beta }}^{\prime }\hat{P}\hat{\beta }}}=\left[\begin{array}{cc} 0.99 & 0.75 \end{array}\right], \end{array}$$

respectively.

#### Estimated DG index parameters

The estimated DG index vector of coefficients was


$${\hat{b}}_{DG}^{\prime }=d^{\prime }(\hat{C}{\hat{P}}^{-1}\hat{C}{)}^{-1}\hat{C}{\hat{P}}^{-1}=\left[-\begin{array}{cc} 26.20 & 216.05 \end{array}\right],$$

from which the estimated expected genetic gain per trait was


$${\hat{E}}_{DG}^{\prime }=(1.755)\frac{{\hat{b}}_{DG}^{\prime }\hat{C}}{\sqrt{{\hat{b}}_{DG}^{\prime }\hat{P}{\hat{b}}_{DG}}}=\left[\begin{array}{cc} -0.60 & 0.60 \end{array}\right],$$

and the estimated DG for the *i*th individual was


$${\hat{I}}_{DG_{i}}=-26.20(Chalk-\bar{Chalk})+21.05(Yield-\bar{Yield}),$$

where $\bar{Chalk}$ and $\bar{Yield}$ denote the average of trait *Chalk* and *Yield*, respectively.

### Selection indices results

#### Economic weight effects on the relationships between traits and indices

Figure 1 is associated with the SIM and shows the relationship between every possible pair of traits Whole, Yield, and SIM for the year 2020 and weights $w^{\prime }=\left[\begin{array}{cc} -1 & 1 \end{array}\right]$ (Fig. 1a) and $w^{\prime }=\left[\begin{array}{cc} -10 & 70 \end{array}\right]$ (Fig. 1b). This figure also shows the relationship between the traits Whole, Ratoon, Yield, and SIM for the year 2021 and weights $w^{\prime }$ = [−1  1  1] (Fig. 1c) and $w^{\prime }$ = [−10  10  70] (Fig. 1d). Furthermore, Fig. 2 shows the relationship between every possible pair of traits Whole, Yield, and ESIM for the year 2020 and weights $F=diag\left\{\begin{array}{cc} -1 & 1 \end{array}\right\}$ (Fig. 2a) and $F=diag\left\{\begin{array}{cc} -10 & 70 \end{array}\right\}$ (Fig. 2b), and shows the relationship between the traits Whole, Ratoon, Yield, and ESIM for the year 2021 and weights ***F*** = *diag*{−1  1  1} (Fig. 2c) and ***F*** = *diag*{−10  10  70} (Fig. 2d). Finally, Fig. 3 shows the relationship between every possible pair of traits Whole, Yield, and DG for the year 2020 and weights $d^{\prime }=\left[\begin{array}{cc} -1 & 1 \end{array}\right]$ (Fig. 3a) and $d^{\prime }=\left[\begin{array}{cc} -10 & 70 \end{array}\right]$ (Fig. 3b) and shows the relationship between the traits Whole, Ratoon, Yield, and DG (the year 2021) for weights $d^{\prime }$ = [−1  1  1] (Fig. 3c) and $d^{\prime }$ = [−10  10  70] (Fig. 3d).

Supplementary Figures 1a–3a show, below the main diagonal, the correlation coefficients with different colors, whereas, above the diagonal, the areas of circles show the absolute value of corresponding correlation coefficients. These figures also show the correlation coefficient of every possible pair of traits Whole, Ratoon, Yield, and the indices for year datasets 2020 and 2021 with 2 sets of trait weights. Note, also, that, for SIM, Supplementary Fig. 1a has 4 sub-figures (Supplementary Fig. 1a–d) which are associated with the trait weights $w^{\prime }=\left[\begin{array}{cc} -1 & 1 \end{array}\right]$ (Supplementary Fig. 1a), $w^{\prime }=\left[\begin{array}{cc} -10 & 70 \end{array}\right]$ (Supplementary Fig. 1b), $w^{\prime }$ = [−1  1  1] (Supplementary Fig. 1c), and $w^{\prime }$ = [−10  10  70] (Supplementary Fig. 1d), respectively. Supplementary Figures 2a and 3a have similar information for ESIM and DG, respectively. Those figures complement Figs. 1–3, and allow a complete interpretation of the trait weight effects on the relationships between traits and indices. Due to this, we shall describe both sets of figures in pairs. That is, Fig. 1 and Supplementary Fig. 1a will be analyzed jointly. Similarly, we will analyze Fig. 2 and Supplementary Fig. 2a, and Fig. 3 and Supplementary Fig. 3a. We will perform this type of analysis because the correlation (Supplementary Figs. 1a, 2a, and 3a) between traits and indices is also shown by the line slope over Figs. 1–3.

The positive correlation between Yield and SIM was 0.93 (Supplementary Fig. 1a) for weights $w^{\prime }=\left[\begin{array}{cc} -10 & 70 \end{array}\right]$ (Supplementary Fig. 1b); whereas for weights $w^{\prime }=\left[\begin{array}{cc} -1 & 1 \end{array}\right]$ (Supplementary Fig. 1a), that correlation was 0.06. Similarly, the positive correlation between Yield and SIM was 0.87 (Supplementary Fig. 1a) for weights $w^{\prime }$ = [−10  10  70] (Supplementary Fig. 1d), whereas for $w^{\prime }$ = [−1  1  1] (Supplementary Fig. 1c), the correlation was 0.28. These correlations are expressed as a linear relationship between trait Yield and the SIM in Fig. 1 (i.e. Fig. 1a–d). This tendency was also observed for ESIM and DG. Thus, the positive correlation between Yield and ESIM (Fig. 2 and Supplementary Fig. 2a) was 1.00 for $F=diag\left\{\begin{array}{cc} -10 & 70 \end{array}\right\}$ (Fig. 2b), whereas for $F=diag\left\{\begin{array}{cc} -1 & 1 \end{array}\right\}$ (Fig. 2a), the correlation was 0.98. Similarly, the positive correlation between Yield and ESIM was 0.85 for weights ***F*** = *diag*{−10  10  70} (Fig. 2d), whereas for ***F*** = *diag*{−1  1  1} (Fig. 2c), the correlation was 0.28. Furthermore, the positive correlation between Yield and DG (Fig. 3 and Supplementary Fig. 3a) was 0.92 for $d^{\prime }=\left[\begin{array}{cc} -10 & 70 \end{array}\right]$ (Fig. 3b), whereas for $d^{\prime }=\left[\begin{array}{cc} -1 & 1 \end{array}\right]$ (Fig. 3a), the correlation was 0.78. Similarly, the positive correlation between Yield and DG was 0.93 for weights $d^{\prime }$ = [−10  10  70] (Fig. 3d), whereas for $d^{\prime }$ = [−1  1  1] (Fig. 3c), the correlation was 0.84. That is, the size of trait weights and DG affect the correlation between the indices and the traits. Thus, because the SIM and ESIM are associated with the net genetic merit, a height correlation of the traits with the indices implies a height correlation with the net genetic merit, whereas in the DG context, a height correlation of traits with DG implies a height correlation with the genetic values.

#### Traits heritability, phenotypic, and genotypic correlations

Table 2 presents the 4 traits (Chalk, Whole, Ratoon, and Yield) broad sense heritabilities, the phenotypic correlations (upper diagonal), genotypic correlations (lower diagonal, underlined), and their standard deviations (in parenthesis) for the years 2018–2022, with a different number of traits and a different number of sample size for 2 trait weights datasets. The standard deviations of the genotypic correlations were obtained according to Roff (1995), whereas the standard deviations of the phenotypic correlations were obtained according to Cerón-Rojas and Crossa (2020, Supplementary Material A). The average of the heritabilities for the 5 years of each trait was 0.463 (Chalk), 0.580 (Whole), 0.436 (Ratoon), and 0.536 (Yield) which means the heritabilities were very similar for all 4 traits. However, note that the averages do not consider years without heritabilities for traits Whole and Ratoon (Table 2), which makes the analysis of heritabilities difficult. We did not obtain the standard deviations of the trait heritability, but this could be obtained according to Lynch and Walsh (1998, p. 818, Eq. A1.19b), among other authors.

**Table 2.** Four traits broad sense heritabilities, phenotypic correlations (upper diagonal, in black), genotypic correlations (down the diagonal, underlined), and their standard deviations (in parenthesis) for the years 2018–2022.

Year	Trait heritabilities				Phenotypic and genotypic correlations for the year 2018			
	Chalk	Whole	Ratoon	Yield	Chalk	Whole	Ratoon	Yield
2018	0.589	0.550	0.294	0.628	1.000	**0.040** (0.086)	**−0.017** (0.086)	**0.236** (0.082)
2019	0.221	0.528	*	0.548	**0.057** (0.428)	1.000	**0.020** (0.086)	**0.288** (0.079)
2020	0.439	*	*	0.511	**0.002** (0.587)	**0.410** (0.505)	1.000	**0.148** (0.084)
2021	0.401	*	0.475	0.467	**0.481** (0.309)	**0.537** (0.296)	**0.546** (0.399)	1.000
2022	0.665	0.663	0.538	0.528				
**Average**	**0.463**	**0.580**	**0.436**	**0.536**				

	Phenotypic and genotypic correlations for the year 2019				Phenotypic and genotypic correlations for the year 2020			
	Chalk	Whole	Ratoon	Yield	Chalk	Whole	Ratoon	Yield
	1.000	**−0.261** (0.051)	*	**−0.014** (0.055)	1.000	*	*	**0.247** (0.054)
	**−0.419** (0.375)	1.000	*	**0.587** (0.036)	*	*	*	*
	*	*	*	*	*	*	*	*
	**−0.086** (0.443)	**0.842** (0.286)	*	1.000	**0.300** (0.31)	*	*	1.000

	Phenotypic and genotypic correlations for the year 2021				Phenotypic and genotypic correlations for the year 2022			
	Chalk	Whole	Ratoon	Yield	Chalk	Whole	Ratoon	Yield
	1.000	*	**−0.101** (0.053)	**0.039** (0.054)	1.000	**−0.412** (0.057)	**−0.251** (0.064)	**0.002** (0.069)
	*	*	*	*	**−0.559** (0.201)	1.000	**0.165** (0.067)	**0.409** (0.057)
	**−0.237** (0.328)	*	1.000	**−0.085** (0.053)	**−0.281** (0.299)	**0.345** (0.287)	1.000	**−0.122** (0.068)
	**0.049** (0.35)	*	**−0.107** (0.318)	1.000	**−0.105** (0.324)	**0.417** (0.271)	**−0.014** (0.364)	1.000

^*^No trait measured for this year.

In most cases, the genotypic correlations and their standard deviations were higher than the phenotypic correlations and standard deviations. This is because the method of estimation of the phenotypic correlations is simpler than the method of estimation of the genotypic correlations. Thus, while the phenotypic correlations can be estimated using the Pearson correlation coefficients, the estimation of the genotypic correlations requires a more complex method (see Holland 2006).

#### Expected genetic gains per traits

Table 3 presents the estimated SIM, ESIM, and DG expected genetic gains per trait obtained within 4 years (2018–2021) with a different number of traits (Chalk, Whole, Ratoon, and Yield) and a different number of sample sizes for 2 trait weights datasets. The interpretation of the results in Table 2 is direct. Thus, positive values of the estimated expected genetic gain per trait indicate that the mean trait genotypic values increase, whereas negative values indicate that such mean values decrease. As indicated earlier, the objective was to decrease the expected genetic gain for trait Chalk, while the other traits increased.

**Table 3.** SIM, ESIM, and DG estimated expected genetic gains per trait for 4 years (2018–2021) and a different number of traits (Chalk, Whole, Ratoon, and Yield) and sample size in each year.

Index	*Year*	Economic weights				Economic weights				Sample size
		−1	1	1	1	−10	10	10	70	
		Chalk	Whole	Ratoon	Yield	Chalk	Whole	Ratoon	Yield	
SIM	2018	−0.789	3.928	0.175	0.417	0.523	4.079	0.215	0.766	134
	2019	−0.97	2.097	*	0.678	−0.648	2.141	*	0.774	332
	2020	−1.85	*	*	0.019	−0.052	*	*	0.699	307
	2021	−1.889	*	0.098	0.098	−0.862	*	0.026	0.462	348
	**Average**	**−1**.**374**	**3.013**	**0**.**137**	**0**.**303**	**−0**.**26**	**3**.**11**	**0**.**121**	**0**.**675**	
ESIM	2018	−0.031	3.643	0.216	0.695	1.082	2.689	0.176	0.831	134
	2019	−0.518	2.126	*	0.787	−0.248	1.87	*	0.782	332
	2020	0.988	*	*	0.746	0.698	*	*	0.755	307
	2021	−0.423	*	−0.19	0.149	−0.168	*	−0.124	0.452	348
	**Average**	**0**.**004**	**2.885**	**0**.**013**	**0**.**594**	**0**.**341**	**2**.**28**	**0**.**026**	**1**.**41**	
DG	2018	−0.162	0.162	0.162	0.162	−0.053	0.053	0.053	0.37	134
	2019	−0.217	0.217	*	0.217	−0.025	0.025	*	0.177	332
	2020	−0.099	*	*	0.692	−0.594	*	*	0.594	307
	2021	−0.198	*	0.198	0.198	−0.069	*	0.069	0.483	348
	**Average**	**−0**.**169**	**0.189**	**0**.**18**	**0**.**317**	**−0**.**185**	**0**.**039**	**0**.**061**	**0**.**406**	

^*^No trait measured for this year.

Except for the averages of the estimated ESIM expected genetic gains associated with Chalk, the averages of the estimated SIM and DG expected genetic gains associated with Chalk were negatives (Table 3), as we would expect. That is, the average of the estimated SIM and DG expected genetic gains associated with Chalk was negative, but positive for ESIM. Nevertheless, note that when the trait weight for Chalk was −1, the estimated ESIM expected genetic gains associated with Chalk were negative for the years 2018, 2019, and 2021, whereas when the trait weight for Chalk was −10, those estimates were negative for 2019 and 2021. This means that the change of sign of the trait weights affected the estimated ESIM expected genetic gain per trait for these datasets differently.

#### Estimated selection response and correlation between the indices and the net genetic merit

Table 4 presents the estimated SIM and ESIM selection response and correlation obtained in 4 years (2018–2021) with different numbers of traits (Chalk, Whole, Ratoon, and Yield), 2 sets of trait weights (**a** and **b**), and a different number of sample size. While for the first dataset of trait weights, the average of the estimated SIM selection responses was higher than the average of the estimated ESIM selection response, the contrary was true for the second dataset. In a similar manner, while the SIM and ESIM average of the estimated correlations for cases (a) were 0.70 and 0.73 for the first dataset of trait weights, for case (b) the SIM and ESIM average were 0.75 and 0.69, respectively. Ceron-Rojas and Crossa (2018, Chapter 7) have described a better method to compare the ESIM and SIM selection response. With that method, the ESIM response can be higher than the SIM selection response.

**Table 4.** SIM and ESIM estimated selection response and correlation between the selection index and the total genetic merit for 4 years (2018–2021) for 2 sets of trait weights (a and b) and a different number of traits (Chalk, Whole, Ratoon, and Yield) and sample size.

Year	SIM		ESIM		Sample size
	Response*^a^*	Correlation*^a^*	Response*^a^*	Correlation*^a^*	
2018	5.31	0.79	1.44	0.78	134
2019	3.74	0.71	2.23	0.79	332
2020	1.87	0.65	1.58	0.72	307
2021	2.09	0.65	0.54	0.64	348
**Average**	**3**.**25**	**0**.**70**	**1**.**45**	**0**.**73**	

Year	SIM		ESIM		Sample size
	Response*^b^*	Correlation*^b^*	Response*^b^*	Correlation*^b^*	
2018	91.36	0.85	70.94	0.68	134
2019	82.06	0.78	100.28	0.69	332
2020	49.43	0.70	103.89	0.72	307
2021	41.23	0.67	9.59	0.67	348
**Average**	**66**.**02**	**0**.**75**	**71**.**17**	**0**.**69**	

^*a*^ $w^{\prime }=\left[\begin{array}{cccc} -1 & 1 & 1 & 1 \end{array}\right]$ (SIM) and $F=diag\left\{\begin{array}{cccc} -1 & 1 & 1 & 1 \end{array}\right\}$ (ESIM).

^*b*^ $w^{\prime }=\left[\begin{array}{cccc} -10 & 10 & 10 & 70 \end{array}\right]$ (SIM) and $F=diag\left\{\begin{array}{cccc} -10 & 10 & 10 & 70 \end{array}\right\}$ (ESIM).

### Genomics relationship matrix expectation

The genomics relationship matrix $G={XX}^{\prime }/\pi$ has special statistical properties. For example, in the asymptotic context the expectation of matrix G is equal to the numerical relationship matrix A, i.e. *E*(G) = A (Van Raden 2008). This means that G is a particular realization of A and when the number of markers and genotypes increases in the population, the value of G tends to concentrate around A. Van Raden (2008) and Isik *et al*. (2017, Chapter 11) have given complete details of matrix G.

### Estimated correlations among the predicted and observed selection index values for the year 2022

Table 5 presents the years of training sets, minimum (Min.), mean, and maximum (Max.) values of the estimated correlations among the predicted and observed selection index values within each year using all negative and positive correlation values and using only positive values obtained with Models 1 and 2 (Table 1). These estimates were obtained with a different number of traits (Chalk, Whole, Ratoon, and Yield) and a different number of sample size for each index (SIM, ESIM, and DG).

**Table 5.** Years of training sets, minimum (Min.), mean, and maximum (Max.) values of the estimated correlations among the predicted and observed selection index values within each year using all negative and positive correlation values and using only positive values obtained with Models 1 and 2.

Year	All data			Positive values only		
	Min.	Mean	Max.	Min.	Mean	Max.
2018	−0.239	−0.131	−0.008	*	*	*
2019	0.309	0.377	0.467	0.309	0.377	0.467
2020	−0.331	0.155	0.366	0.251	0.317	0.366
2021	−0.299	0.000	0.140	0.070	0.100	0.140
2020–2021	−0.301	0.104	0.281	0.204	0.240	0.281
2018–2019–2020	−0.025	0.228	0.343	*	*	*
2019–2020–2021	0.051	0.292	0.397	0.051	0.301	0.397
**Average**	**−0**.**119**	**0**.**147**	**0**.**284**	**0**.**177**	**0**.**267**	**0**.**330**

Year	All data			Positive values only		
	Min.	Mean	Max.	Min.	Mean	Max.
2018	−0.252	−0.140	0.024	0.024	0.024	0.024
2019	0.317	0.385	0.459	0.317	0.385	0.459
2020	−0.328	0.157	0.374	0.242	0.318	0.374
2021	−0.312	0.001	0.144	0.078	0.105	0.144
2020–2021	−0.311	0.107	0.295	0.202	0.246	0.295
2018–2019–2020	−0.025	0.229	0.348	*	*	*
2019–2020–2021	0.043	0.293	0.411	0.043	0.302	0.411
**Average**	**−0**.**124**	**0**.**147**	**0**.**293**	**0**.**151**	**0**.**230**	**0**.**284**

^*^No correlation measured for this year.

#### General trends across Models 1 and 2

Table 5 provides an overview of the correlations between the predicted and observed selection index values for Models 1 and 2 across various training sets and subsets of correlation values. The average values of minimum, mean, and maximum correlations for Models 1 and 2 are comparable, proving that the inclusion of environmental covariates in Model 2 does not significantly impact the overall prediction of index values. Excluding negative correlation values enhances the average minimum and mean correlations for both models, with Model 1 showing a slightly higher performance in this context. Certain years, particularly 2019 and combined multi-year datasets (e.g. **2019–2020–2021**), yield higher correlations, suggesting that they provide more reliable or representative training data, while environmental covariates slightly improve the average of the maximum correlation values (e.g. 0.284 in Model 1 vs 0.293 in Model 2). In a similar manner, their overall effect on the average of mean and minimum correlations is minimal.

#### General trends across Models 3–5

Table 6 presents the years of training sets, minimum (Min.), mean, and maximum (Max.) values of the estimated correlations among the predicted and observed selection index values within each year using all negative and positive correlation values and using only positive values obtained with Models 3–5. For all 3 models, the average of the minimum (Min.), mean, and maximum (Max.) values of the estimated correlations among the predicted and observed selection index values within each year were very similar. This means that Model 3 is adequate for the prediction of the index values. In addition, note that the average of the maximum values of Models 3–5 were higher than the average of the maximum values of Models 1 and 2. Thus, the covariable $\beta_{Y}Year_{ij}$ is a good option to incorporate in the model predictions.

**Table 6.** Years of training sets, minimum (Min.), mean, and maximum (Max.) values of the estimated correlations between the predicted and observed selection index values within each year using all negative and positive correlation values and using only positive values obtained with Models 3–5.

	All data			Positive Values only		
Year	Min.	Mean	Max.	Min.	Mean	Max.
2020–2021	−0.318	0.109	0.297	0.217	0.251	0.297
2018–2019–2020	−0.023	0.228	0.339	*	*	*
2019–2020–2021	0.049	0.294	0.403	0.049	0.302	0.403
**Average**	**−0**.**097**	**0**.**210**	**0**.**346**	**0**.**133**	**0**.**276**	**0**.**350**

	All data			Positive values only		
Year	Min.	Mean	Max.	Min.	Mean	Max.
2020–2021	−0.317	0.109	0.300	0.211	0.251	0.300
2018–2019–2020	−0.020	0.232	0.354	*	*	*
2019–2020–2021	0.048	0.295	0.411	0.048	0.304	0.411
**Average**	**−0**.**097**	**0**.**212**	**0**.**355**	**0**.**130**	**0**.**277**	**0**.**356**

	All data			Positive values only		
Year	Min.	Mean	Max.	Min.	Mean	Max.
2020–2021	−0.314	0.112	0.306	0.215	0.253	0.306
2018–2019–2020	−0.020	0.234	0.357	*	*	*
2019–2020–2021	0.051	0.296	0.406	0.051	0.305	0.406
**Average**	**−0**.**095**	**0**.**214**	**0**.**356**	**0**.**133**	**0**.**279**	**0**.**356**

^*^No data for these years.

Models 3–5 yield similar results, with slight improvements observed as additional covariates are included (from Model 3 to Model 5). The addition of covariates in Models 4 and 5 enhances prediction performance slightly, as reflected in higher maximum and mean correlation values compared with Model 3. Filtering for positive values increases the minimum and mean correlations across all models, improving the robustness of the predictions.

Training sets for **2019–2020–2021** consistently provide the highest correlations, indicating that this training set captures relevant variability or relationships for prediction. The year **2020–2021**, on the other hand, shows weaker correlations, likely due to limitations in data representativeness or prediction conditions for these years. Among the 3 models, Model 5 shows the best overall performance, particularly in terms of maximum correlation values, suggesting that it is the most reliable for selection index predictions.

#### General trends across Models 1–5

Model 2 (environmental covariates) and Models 3–5 (additional covariates) perform slightly better than Model 1 in terms of mean and maximum correlation values. Models 4 and 5 show the highest correlations, suggesting that the inclusion of covariates ($\beta_{Y}Year_{ir}$ and $g_{YCovs_{ir}}$) is beneficial for prediction accuracy. Model 5 consistently achieves the highest average maximum correlations (0.356 for both all data and positive values only).

Training sets that span multiple years (e.g. **2019–2020–2021**) provide better predictions across all models, likely due to the inclusion of diverse and representative data. However, single-year datasets (e.g. **2020–2021**) often yield lower correlations, reflecting limited or less representative training data.

In summary, removing negative correlations improves minimum and mean values across all models, making predictions more robust. Models incorporating additional covariates (Models 3–5) outperform those without them (Models 1 and 2) in terms of mean and maximum correlations. The most robust and accurate predictions are achieved by **Model 5**, particularly for datasets spanning multiple years, such as **2019–2020–2021**. Including covariates enhances predictive performance, particularly for maximum correlations. Positive correlations alone provide a clearer picture of model performance, with improved minimum and mean values across all models.

#### ESIM and SIM correlations between the observed and predicted values

Table 7 presents the ESIM, SIM, trait weights (***w*, *F***), total training (year/s), environmental covariables (EC) used in model predictor [*Year*, *G*, EC measures similarity between year(s) measured as kernel (*K*) linear (*L*), or kernel Gaussian (*KG*)], *YearCovsKL* = year covariables measure as liner kernel (*KL*) or as *YearCovsKG* as Gaussian kernel, and the estimated top 30 correlations (Cor) between the estimated (4 years: 2018–2021) and predicted (for the year 2022) index values. For these datasets, ESIM and SIM estimates were the best values to predict the index values in the year 2022.

**Table 7.** ESIM, SIM, trait weights (*w*, *F*), total training (year/s), EC used in the model predictor [*Year*, *G*, EC measures similarity between year(s) measured as kernel (*K*) linear (*L*) or kernel Gaussian (*KG*)], *YearCovsKL* = year covariables measure as linear kernel (*KL*) or as *YearCovsKG* as Gaussian kernel, and the estimated top 30 correlations (*Cor*) between the estimated (4 years: 2018–2021) and predicted (for the year 2022) index values, ranking in descending order.

ESIM, SIM, and trait weights	Total training (year/s)	EC	Model predictor	*Cor*
ESIM_$F_{2}$	2019	NA	1 (G)	0.4667
ESIM_$F_{2}$	2019	Average of training years	2 (G + Year Env. Cov. Linear)	0.4585
ESIM_$F_{2}$	2019, 2020, 2021	Of two years ago	4 (Year Resp + G + Year Cov. Linear)	0.4108
ESIM_$F_{2}$	2019, 2020, 2021	Of previous year	2 (G + Year Env. Cov. Linear)	0.4107
ESIM_$F_{2}$	2019, 2020, 2021	Average of training years	4 (Year Resp + G + Year. Cov. Linear)	0.4090
ESIM_$F_{2}$	2019, 2020, 2021	Of previous year	4 (Year Resp + G + Year. Cov. Linear)	0.4083
ESIM_$F_{2}$	2019, 2020, 2021	Average of training years	5 (Year Resp + G + Year.Cov. Gaussian)	0.4065
ESIM_$F_{2}$	2019, 2020, 2021	Of previous year	5 (Year Resp + G + Year.Cov. Gaussian)	0.4062
ESIM_$F_{2}$	2019, 2020, 2021	Average of training years	2 (G + Year Env. Cov. Linear)	0.4056
ESIM_$F_{2}$	2019, 2020, 2021	Of two years ago	4 (Year Resp + G + Year. Cov. Linear	0.4027
ESIM_$F_{2}$	2019, 2020, 2021	NA	3 (Year Resp + G)	0.4026
SIM_***w***_2_	2019, 2020, 2021	Of previous year	5 (Year Resp + G + Year.Cov.Gaussian)	0.3995
ESIM_$F_{2}$	2019, 2020, 2021	Of two years ago	2 (G + Year Env. Cov. Linear)	0.3985
SIM_***w***_2_	2019, 2020, 2021	Average of training years	5 (Year Resp + G + Year.Cov.Gaussian)	0.3978
ESIM_$F_{2}$	2019, 2020, 2021	NA	1(G)	0.3974
SIM_**w**_2_	2019, 2020, 2021	Average of training years	4 (Year Resp + G + Year. Cov. Linear)	0.3959
SIM_***w***_2_	2019, 2020, 2021	Average of training years	2 (G + Year Env. Cov. Linear)	0.3954
SIM_***w***_2_	2019, 2020, 2021	Of two years ago	4 (Year Resp + G + Year. Cov. Linear)	0.3954
SIM_***w***_2_	2019, 2020, 2021	NA	3 (Year Resp + G)	0.3952
ESIM_$F_{1}$	2019	Average of training years	2 (G + Year Env. Cov. Linear)	0.3931
SIM_***w***_2_	2019, 2020, 2021	Of two years ago	2 (G + Year Env. Cov. Linear)	0.3930
SIM_***w***_2_	2019, 2020, 2021	Of previous year	2 (G + Year Env. Cov. Linear)	0.3927
SIM_***w***_2_	2019, 2020, 2021	NA	1 (G)	0.3906
SIM_***w***_2_	2019, 2020, 2021	Of previous year	4 (Year Resp + G + Year. Cov. Linear)	0.3885
SIM_***w***_2_	2019, 2020, 2021	Of two years ago	5 (Year Resp + G + Year. Cov. Gaussian)	0.3884
ESIM_$F_{2}$	2020	Average of training years	2 (G + Year Env. Cov. Linear)	0.3736
SIM_***w***_2_	2019	Average of training years	2 (G + Year Env. Cov. Linear)	0.3725
ESIM_$F_{1}$	2019	NA	1 (G)	0.3688
ESIM_$F_{2}$	2020	NA	1 (G)	0.3655
SIM_***w***_2_	2019	NA	1 (G)	0.3645

$F_{1}=diag\left\{\begin{array}{cccc} -1 & 1 & 1 & 1 \end{array}\right\}$ ,$F_{2}=diag\left\{\begin{array}{cccc} -10 & 10 & 10 & 70 \end{array}\right\}$, and $w_{2}^{\prime }=\left[\begin{array}{cccc} -10 & 10 & 10 & 70 \end{array}\right]$.

Years underlined used for the prediction year 2022.

Correlations in Table 7 are in decreasing order. Note that the rank between the correlations associated with the SIM is 0.035, whereas the rank between the correlations associated with the ESIM is 0.074. The top estimated correlation associated with the SIM was 0.3995, whereas the top estimated correlation associated with the ESIM was 0.4667. In a similar manner, the bottom estimated correlation associated with the SIM was 0.3645 and the bottom estimated correlation associated with the ESIM was 0.3655. That is, the top and bottom estimated correlations associated with the ESIM were higher than for the SIM. In addition, both indices were mainly associated with the trait weights −10, 10, 10, 70. The models associated with the prediction index values in Table 6 were mainly Models 1, 2, 4, and 5. That is, in this case, Model 3 is out of the estimated top 30 correlations (Cor) between the estimated (4 years: 2018–2021) and predicted (for the year 2022) index values.

## Discussion

In this work, we evaluated the efficiency of the SIM (Smith 1936) and ESIM (Ceron-Rojas *et al*. 2008) selection indices to predict the net genetic merit, to estimate the selection response, correlation, and the expected genetic gain and, also, we evaluated the efficiency of the DG (Pesek and Baker 1969) to estimate the expected genetic gain per trait using real rice (*O. sativa*) historical datasets (2018–2022). This study provides one of the first comprehensive evaluations of genomic prediction applied directly to multi-trait selection indices—SIM, ESIM, and DG—using historical field data from a rice breeding program. It highlights how different index formulations influence response to selection, predictive ability, and trait interrelationships under realistic breeding conditions. By integrating genomic models, environmental covariates, and economically meaningful trait weights, the research advances the practical implementation of index-based selection. While the results demonstrate that the SIM and ESIM performed more robustly than DG in this rice dataset, we acknowledge that the relative utility of these indices may vary across crops, depending on genetic architecture, trait correlations, and breeding objectives. Nonetheless, the methodology and insights offered here provide a valuable framework that can be adapted to other breeding programs.

The incorporation of environmental covariables prevailing during growth conditions into genomic prediction models of various selection indices provides valuable insights that enhance the prediction accuracy for rice lines across different growth stages. Integrating environmental information allows for capturing genotype-by-environment interactions more effectively, leading to more precise predictions and improved breeding decisions. This approach leverages environmental variation to refine selection strategies, ultimately facilitating the identification of rice lines better adapted to specific environmental conditions, thus accelerating genetic gains and cultivar development in rice breeding programs (Jarquín *et al*. 2014; Millet *et al*. 2019).

The main objective of the SIM and ESIM was to predict the net genetic merit, which is a linear combination of unobservable additive genotypic values weighted by the trait economic values. Nonetheless, the main objective of the DG was to estimate the mean of the breeding values associated with each trait. This means that while the DG index quantified its breeding objectives regarding desired genetic gains (Werner *et al*. 2024), the SIM should reflect the costs and returns involved in a *production* system (Kinghorn 2005).

In the SIM context, economic weight is the increase in profit achieved by improving a particular trait by 1 unit and should reflect the market situation and not only preferences or arbitrary values (Hazel 1943; Magnussen 1990; Charffeddine and Alenda 1998; Blasco 2021). For this reason, the SIM requires a profit function (net returns minus costs) to derive trait economic weights to predict the net genetic merit. However, in plant breeding, e.g. in maize (*Zea mays* L.) and wheat (*Triticum aestivum*) breeding, only grain yield has a specific market price, which makes the application of a profit function very difficult (Ceron-Rojas *et al*. 2023).

The estimated expected genetic gain per trait obtained using rice (*O. sativa*) historical data (2018–2022), indicated that the SIM and ESIM were, generally, higher than the estimated expected genetic gain per trait of DG. However, since the index results depend on factors such as breeding objectives, accuracy of the estimated genetic parameters, trait economic weights, breeder desired genetic gains, and resource constraints, the results of this work might only be generalized to some possible selection programs. Nevertheless, the result of this work indicates that the SIM and ESIM may be a better selection tool in breeding programs than the DG index.

In the SIM context, it is assumed that there is a set of known trait economic weights and that this vector maximizes the correlation between SIM and the net genetic merit and the selection response. In turn, in ESIM, there is not a set of known trait economic weights. Nevertheless, the ESIM vector of coefficients maximizes the correlation between the ESIM and the net genetic merit and the selection response, but because that vector is normalized, the estimated ESIM selection response can be lower than the estimated SIM selection response.

Cerón-Rojas and Crossa (2018, Chapter 7) have indicated that the best way to compare the estimated SIM selection response vs the estimated ESIM selection response occurs when the estimated SIM vector of coefficients is normalized. In this case, the estimated ESIM selection response can be higher than the estimated SIM selection response. In addition, since the correlations of both indices with the net genetic merit are invariant to scale change, the size of this parameter is an adequate tool to compare the efficiency of both indices to predict the net genetic merit. In this work, the SIM and ESIM correlations with the net genetic merit were very similar.

The DG index does not use economic weights; hence, this index does not predict the net genetic merit, nor does it allow to estimate the selection response or the correlation between the DG and the net genetic merit. In this case, it is only possible to estimate the expected genetic gain per trait, that is, the average of breeding values associated with each trait. This means that in the SIM context, the DG index is only a partial index that estimates only a SIM parameter.

### Selection indices main parameters

While the SIM and ESIM were evaluated with 3 parameters (selection response, correlation between indices and the net genetic merit, and expected genetic gain per trait), the DG index was assessed only through the expected genetic gain per trait. This is an evident problem because how to compare the DG index with the others only with 1 parameter? Might the DG be better in this condition than the other 2 indices? Some authors (Werner *et al*. 2024) have indicated that the DG index is optimum and, according to these authors, when the DG vector of coefficients is equal to the SIM vector of coefficients, the DG efficiency is the same as the SIM efficiency, which is evident because in such a case, the DG index and the SIM are the same.

More problems are associated with the estimated expected genetic gains per trait than the estimated selection response and correlation. For example, while the statistical sampling properties of the SIM and ESIM estimators of the selection response and correlation are known in the asymptotic context (Cerón-Rojas and Crossa 2018, 2020, 2022), the sampling properties of the estimator of the expected genetic gain per trait are unknown. Cerón-Rojas and Crossa (2020) have shown that the estimators of the estimated SIM selection response and correlation are unbiased and their variance tends to zero in the asymptotic context.

An additional problem related to the expected genetic gains per trait is that nobody has developed a complete theory of this parameter. In Supplementary Material B, we briefly review the Kempthorne and Nordskog (1959) theory associated with this parameter. These authors defined the expected genetic gains per trait in the restricted index selection context, but they did not investigate the statistical properties of the estimator of this parameter. We would expect that the maximum likelihood estimator of the expected genetic gain per trait is unbiased and that its variance tends to zero in the asymptotic context. However, this is only a conjecture, which is necessary to show.

### Indices correlation and selection response

The correlation between SIM and *H* reflects how accurately the index predicts the true net genetic merit value of individuals. A higher correlation indicates that the index captures more of the genetic variation in the target traits, leading to a more efficient selection. This criterion is critical, since the goal of a selection index is to serve as a reliable proxy for the net genetic merit, ensuring that selected individuals will have a superior overall performance (Hazel 1943).

The response to selection measures the realized genetic improvement in a population after selection is applied. This criterion accounts for how well the selection index performs in practice, considering factors such as genetic variances, covariances, and the heritability of traits. A strong response to selection indicates that the index effectively prioritizes individuals who contribute most to genetic progress in the breeding program (Falconer and Mackay 1996). Together, the correlation and selection response ensure that selection indices are not only theoretically sound but also practically effective in achieving the desired genetic improvements.

### Expected genetic gains per trait

The expected genetic gain per trait evaluates how well the selection index balances improvements across multiple traits. Breeders often deal with traits that have antagonistic relationships, such as yield and disease resistance. A well-constructed index should achieve gains in all desirable directions while adhering to the breeder's objectives. This criterion helps assess whether the index meets specific breeding goals, such as improving one trait without excessively compromising others.

### Trait weights and desired gains

The weights used in this work have 3 possible interpretations: (1) in the SIM context, they are trait economic weights (***w***); (2) in the ESIM context, they are only trait weights (no economic weight) which are used in the *transforming matrix*  F, which according to the similar matrix's theory (Harville 1997; Watkins 2002), it allows us to change the direction of the eigenvectors without affecting the correlation between ESIM and the net genetic merit; and (3) in the DG context, they are desired gains (***d***) imposed by breeders.

In point (1), vector ***w*** is the increase in profit (net returns minus costs) achieved by improving a particular trait by 1 unit, while the others remain fixed (Charffeddine and Alenda 1998; Blasco 2021). In this case, ***w*** should reflect the market situation and the marginal benefit from 1 unit of improvement, as opposed to just preferences or arbitrarily fixed values (Magnussen 1990).

In point (2), the vector of weights ($w_{E}$) do not reflect the market situation or the marginal benefit from 1 unit of improvement. However, they are not arbitrarily fixed values, since these values maximize the correlation between the ESIM and the net genetic merit. In this case, the weights are a linear combination of the first eigenvector (β) of the multi-trait heritability matrix ($P^{-1}C$), that is, $w_{E}=C^{-1}P\beta$, and along with β, maximize the correlation between the ESIM and the net genetic merit, as indicated earlier. That is, $w_{E}$ is different to ***w***, but both vectors should maximize the correlation between the indices and the net genetic merit.

In point (3), we do not have a vector of weights, but a vector of DG (d) given by the breeders. In this case, d does not maximize the correlation between the DG and the net genetic merit, but only constrains the possible values of the expected genetic gain per trait (Eq. 7). Kinghorn (2005) indicated that, in practice, the estimated expected genetic gain per trait is never equal to vector d, as we have observed in this work. Cerón-Rojas and Crossa (2018, Chapter 2) indicated that the DG index is similar to the Tallis (1962, 1985) restriction $U^{\prime }Cb=\theta d$ for $U^{\prime }=I$ and θ=1. The difference between the DG and the Tallis (1962, 1985) index is that the latter index uses economic weight and maximizes the correlation with the net genetic merit with some restrictions.

The above 3 points indicate that the interpretation of the results of the 3 indices is not simple. The results have an economic interpretation in the first case, whereas in the last 2 cases, the interpretations are only associated with genetic gain. Nevertheless, we compared the estimated parameter values of the indices in this work without making the 3 differences described in this subsection.

### The economic weights problem

Economic weights are essential for SIM as they determine the relative importance of traits based on their contribution to profitability. Optimally assessing these weights should consider defining breeding goals by identifying traits that impact the economic performance (e.g. yield, disease resistance) based on the production system and market demands. SIM calculates economic values using partial budget analysis to estimate profit or cost changes per unit of change in each trait and incorporate market prices, input costs, and risk factors.

In addition, it is important to incorporate genetic parameters using heritability genetic variances, and trait correlations to ensure that weights align with achievable genetic gains. It is advisable to use optimization models and simulations to refine weights under different scenarios, validate weights with historical data, and adjust based on stakeholder feedback and changing conditions and update weights as markets, production systems, or environmental conditions evolve. This approach ensures that economic weights drive genetic progress toward maximum profitability while considering practical and genetic constraints.

Cerón-Rojas *et al*. (2023) emphasized the untapped potential of developing nonlinear profit functions for deriving economic weights, an approach that could significantly enhance selection strategies in plant breeding. Their research represents an innovative attempt to formally integrate economic weights into selection indices, providing a framework to improve the precision and efficiency of decision-making in breeding programs. The authors specifically designed a profit function tailored for maize and wheat breeding programs by extending Smith's concept and using linear regression theory. This approach bridges the gap between theoretical advancements and practical breeding applications. In contrast to animal breeding programs, where market prices are assigned to all economic traits of interest, maize and wheat breeding programs typically attribute a market price only to grain yield. As a result, the proposed profit function is structured into 2 distinct components: one directly associated with grain yield and the other representing the expected grain yield based on the performance of additional traits.

By applying this methodology, Ceron-Rojas *et al*. (2023) observed an average estimated correlation of 0.820 between the SIM and the net genetic merit across 7 simulated selection cycles. For real datasets, the correlation values were even stronger, reaching 0.87 and 0.85 for maize and wheat, respectively.

### The genomic prediction models

In Table 7, the 10 top predictor models were numbers 1, 2, 4, and 5. These models included environmental covariables and years in the prediction of unobserved index values. Nevertheless, Table 7 indicates that the 10 bottom predictor models were numbers 1, 2, 4, and 5. That is, Models 4 and 5 also appeared, which included a higher number of covariables. Note, however, that in Table 7, the bottom 5 models were only 1 and 2, which include a few numbers of covariables. These results indicate that the prediction of unobservable index values using historical data is not a simple task and that additional investigation in this area is necessary.

The results from Tables 6 and 7 highlight critical insights into the performance and utility of different prediction models for the prediction of selection index values. Across the evaluated models, key takeaways emerge:

#### Prediction accuracy depends on model complexity but with diminishing returns

Models 2, 3, 4, and 5, which incorporate additional covariates, generally outperform Model 1. However, the differences in prediction accuracy are modest, indicating that the increasing model complexity does not always lead to proportionate improvements. For example, the performance of Model 3, which is less complex, is nearly as strong as in Models 4 and 5, suggesting that simpler models can be effective under certain conditions.

#### Positive correlation values are more informative

Filtering to include only positive correlation values consistently improves mean and maximum prediction accuracy across all models. This suggests that excluding negative correlations is an effective strategy for focusing on meaningful predictive relationships.

Positive-value filtering highlights the predictive potential of these models in scenarios where favorable associations are prioritized.

#### Training data diversity and size matter

Models trained on larger and more diverse datasets spanning multiple years (e.g. 2019–2020–2021) exhibit higher prediction accuracies compared with those trained on single-year data. This underscores the importance of comprehensive and representative datasets in prediction model training.

#### Robustness across years varies

There is variability in model performance across years, with some years (e.g. 2019) showing stronger correlations, whereas others (e.g. 2018 and 2021) reveal weaker associations. This variability reflects potential environmental or dataset-specific influences that impact prediction accuracy.

#### The core message

The findings emphasize that while increasing model complexity and incorporating additional covariates can enhance prediction accuracy, these improvements are often incremental. Simpler models, like Models 1–3, can perform comparably well under the right conditions, making them attractive options for practical applications where computational efficiency or interpretability is critical.

Furthermore, the results highlight the importance of thoughtful data selection and preparation. Focusing on positive correlations and leveraging diverse, multi-year datasets can significantly enhance prediction reliability. This provides valuable guidance for optimizing prediction model design and data strategies in genomic prediction studies.

#### Why was the correlation between the estimated DG values and their predicted values very low?

Results presented in Table 7 indicate that the ESIM was the best predictor of estimated index values in 2022. However, why was the correlation between the estimated DG values and their predicted values very low? Three explanations are possible: (1) the definition of the DG index does not allow to predict indices values, (2) the estimation of the DG index vector of coefficients might be not accurate, and (3) since the DG index only allows us to estimate the mean values of the traits, and does not allow us to predict the net genetic merit (Eq. 3), the DG index is not able to predict linear combination of traits, that is, selection index values.

#### The DG index is not clearly defined

We analyze point (1) based on Supplementary Material B (Eq. 1), where we have described the expected genetic gain per trait theory of Kempthorne and Nordskog (1959). The SIM is the best linear predictor of *H* in the phenotypic context, that is, it is the conditional expectation of the net genetic merit given the phenotypic record (y) written as


$$I_{i}=E(H_{i}|y_{i})=[\mathrm{var}(y_{i})]{-}^{1}\mathrm{cov}(H_{i},y_{i})(y_{i}-\mu_{i})=P^{-1}Cw(y_{i}-\mu_{i}),$$

where $\mu_{i}$ is the mean vector of $y_{i}$, $[\mathrm{var}(y_{i}){]}^{\ddot{-}1}=P^{-1}$, and $\mathrm{cov}(H_{i},y_{i})=Cw$. It is not possible to obtain a similar definition for DG. In the DG context, the definition of the DG index is included in the definition of the expected genetic gain per trait (Eq. 7) and is not *clearly defined*, so it can be any 1 index, except ESIM or SIM.

The DG vector of coefficients (Eq. 11) indicates that this index is not the best predictor of the net genetic merit; hence, we have an undetermined index where such an index might not be optimum. Itoh and Yamada (1986) minimized $b^{\prime }Pb$ under the restriction Cb=d, and they obtained the DG vector of coefficients. This implies that DG and SIM are different except in the unlikely case in which the vector of coefficients is the same in both indices (Cotterill and Jackson 1985; Werner *et al*. 2024).

#### Conditional expectation of SIM and DG

We would expect that the conditional expectation of SIM given the net genetic merit be near Eq. (3) in the asymptotic context. It is possible to show that


$$E(I_{i}|H_{i})=\rho_{HI}^{2}H_{i},$$

or in other words, the SIM is a proportion $\rho_{HI}^{2}$ of $H_{i}=w^{\prime }c_{i}$, hence, when $\rho_{HI}$ tends toward 1.0, the SIM values tend to $H_{i}=w^{\prime }c_{i}$, as we should expect. Similarly, it is possible to show that the expectation of DG given $c_{i}$ is


$$E(DG|c_{i})=b_{DG}^{\prime }c_{i}.$$

In other words, DG is not a propotion $\rho_{HI}^{2}$ of $H_{i}=w^{\prime }c_{i}$ (Eq. 3). In the latter case, we would expect that the best prediction of DG be when $b_{DG}^{\prime }$ is a vector of ones, but, in general, this is not the case.

#### The DG does not minimize the mean square error

Another problem with DG is that this index does not minimize the mean square error between $c_{i}$ and DG because it is impossible to define the mean square error in the DG context. Note that the mean square error in the SIM context is defined as


$$E[{\left(H_{i}-I_{i}\right)}^{2}],$$

which is usually minimized concerning the SIM vector of coefficients (Cerón-Rojas and Crossa 2018, Chapter 2) to maximize the selection response. But how to define the mean square error between a vector ($c_{i}$) and a scalar, as should be in the DG context?

#### Additional DG problems

The relatively lower performance of the DG index in our analyses can be attributed to several conceptual and practical limitations. Unlike SIM and ESIM, the DG index requires users to specify fixed target values for genetic gains in each trait. While this can be useful in theory, these desired gains may not reflect biologically realistic outcomes given genetic correlations or selection constraints. Moreover, the DG index relies on the inverse of the genetic covariance matrix, which can lead to instability or extreme weight estimates, particularly when the matrix is ill-conditioned. Compared with the SIM, which incorporates economic weights and allows for more nuanced trade-offs between traits, the DG index is less flexible and lacks an underlying optimization framework that maximizes the total genetic merit. Additionally, because the DG method enforces gain constraints rather than maximizing an aggregate index, it may select individuals that meet the predefined targets but do not optimize the overall breeding value. These limitations help explain the reduced robustness of the DG index across years and environments in our study.

## Conclusion

This study demonstrated that the SIM and ESIM selection indices effectively predict the net genetic merit, estimate selection response, and compute correlation with the net genetic merit, whereas the DG index only defines and estimates the expected genetic gain per trait. We highlighted key limitations of the DG index and concluded that SIM could be a superior selection tool compared with DG in breeding programs. Specifically, SIM was highly efficient at maximizing the selection response, correlation, and expected genetic gain per trait, while ESIM and DG showed strong performance under certain noneconomic conditions. This underscores the potential benefits of integrating genetic and environmental information in selection decisions. Additionally, we evaluated 5 genomic prediction models to predict future selection index values using historical datasets. Results indicated that prediction models need not be overly complex; however, genomic prediction models (Models 3–5) incorporating environmental covariates significantly enhanced the accuracy of predicting unobserved selection indices using ESIM and SIM.

## Supplementary Material

jkaf087_Supplementary_Data

## Data Availability

The datasets for 5 years (2018, 2019, 2020, 2021, and 2022) with real rice datasets had different number of cultivars and traits in each year [traits included are Chalk (%), Whole milling (%), Ratoon yield (ton/h), and Yield grain (ton/h)] to evaluate and compare the SIM, ESIM, and DG efficiency and to predict the SIM, ESIM, and DG values for the year 2022. These datasets are in the link https://github.com/GHAML1/GPSI-EH-091124. Supplemental material available at G3 online.
