# Supplementary material for: Evaluating the effectiveness of selection indices and their genomic prediction using environmental and historical rice data
Source: G3 (Bethesda). 2025 Apr 16;15(6):jkaf087. doi: 10.1093/g3journal/jkaf087 (PMC12135014; doi:10.1093/g3journal/jkaf087)
Supplement: jkaf087_Supplementary_Data [file jkaf087_supplementary_data.docx]

**Evaluating the Effectiveness of Selection Indices and their Genomic Prediction Using Environmental and Historical Rice Data**

**Supplementary Material A**

**Figures S1A**, **S2A**, and **S3A** show the correlation coefficient of every possible pair of traits Chalk, Whole, Ratoon, Yield, and the indices for year datasets 2020 and 2021 with two sets of trait weights. Note, also, that for each index (SIM. ESIM, and DG) each figure has four sub-figures (Figs. A, B, C, and D) which are associated to the trait weights of each index.


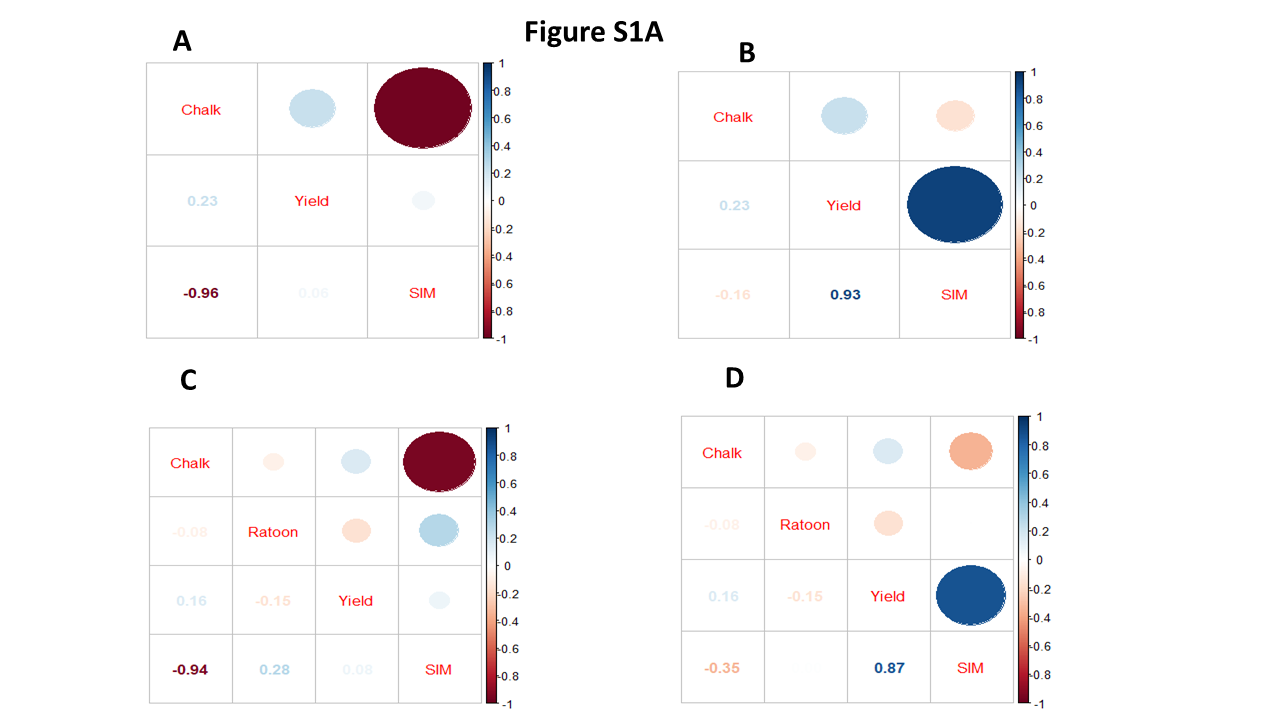


**Figure S1A** Correlations among traits Whole, Yield, and SIM (Smith Index Method, year 2020) for weights $\mathbf{w}'=\left[\begin{matrix} -1 & 1 \end{matrix}\right]$(Fig. A) and $\mathbf{w}'=\left[\begin{matrix} -10 & 70 \end{matrix}\right]$(Fig. B); correlations among traits Whole, Ratoon, Yield, and SIM (year 2021) for weights **w** = [-1  1  1] (Fig. C) and **w** = [-10  10  70] (Fig. D).


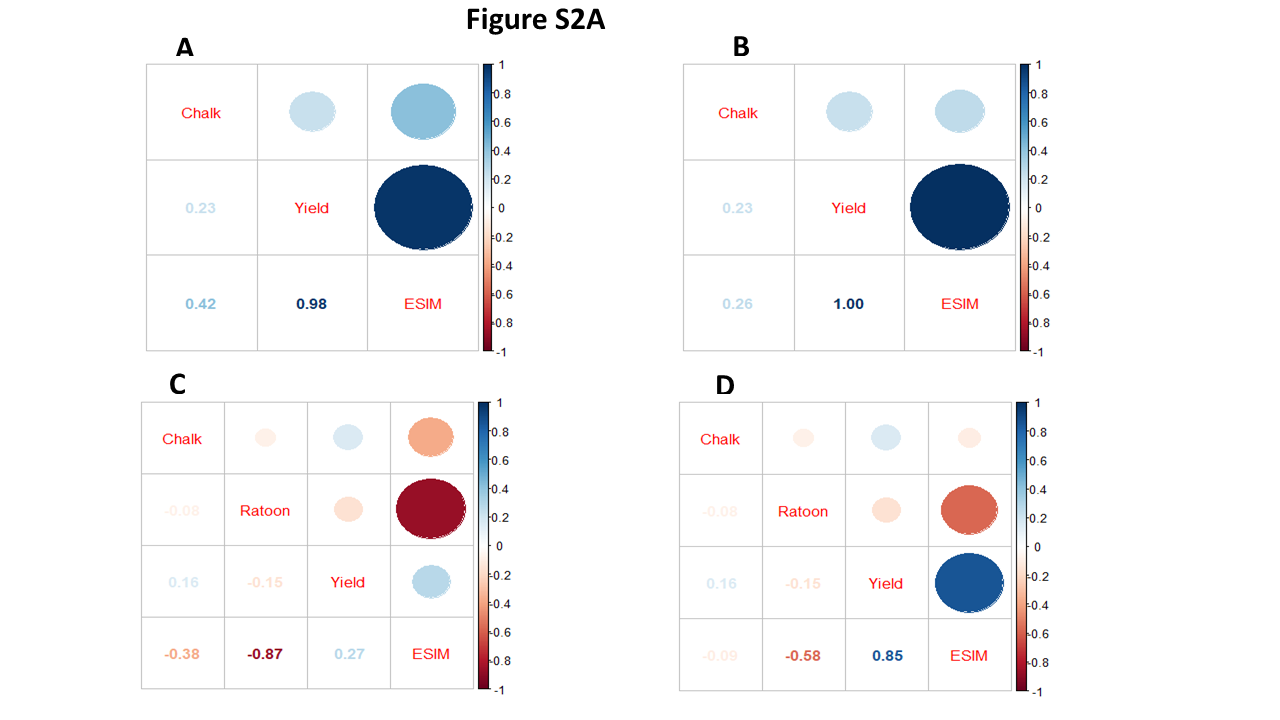


**Figure S2A** Correlations among traits Whole, Yield, and ESIM (Eigen Selection Index Method, year 2020) for weights $\mathbf{F}=diag\left\{\begin{matrix} -1 & 1 \end{matrix}\right\}$ (Fig. A) and $\mathbf{F}=diag\left\{\begin{matrix} -10 & 70 \end{matrix}\right\}$ (Fig. B); correlations among traits Whole, Ratoon, Yield, and ESIM (year 2021) for weights **F** =*diag*{-1  1  1} (Fig. C) and **F** = *diag*{-10  10  70} (Fig. D).


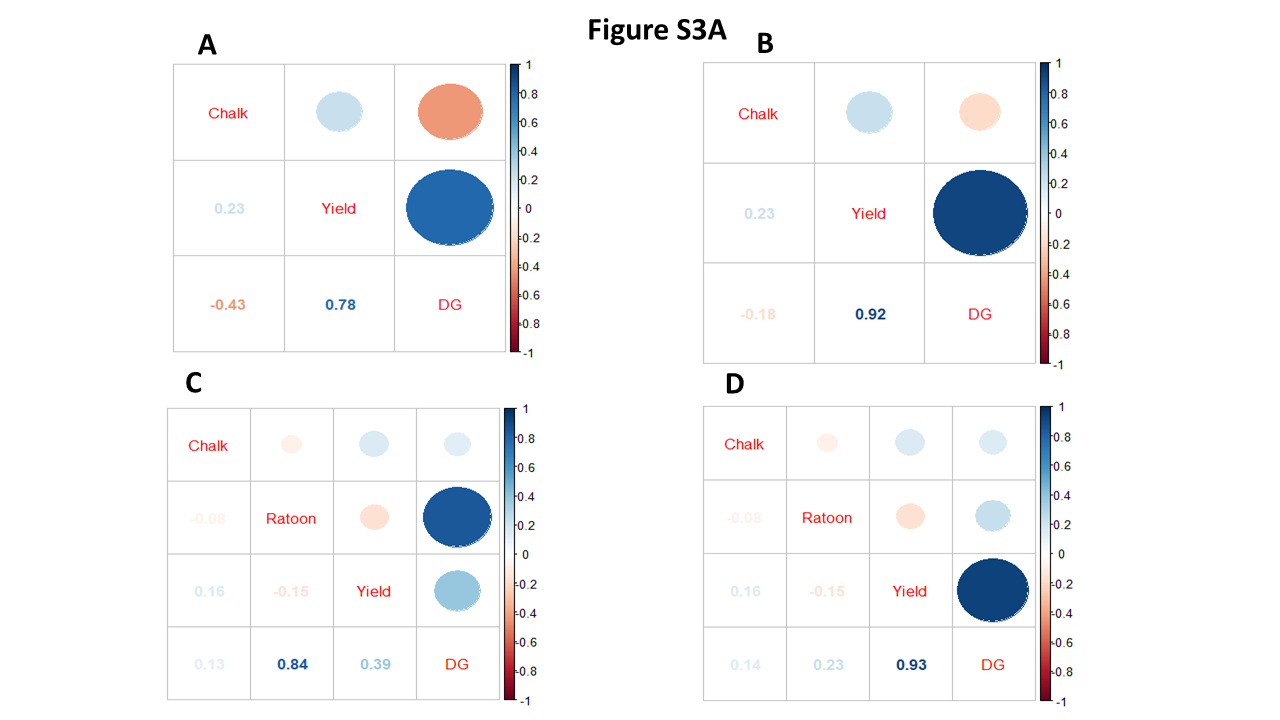


**Figure S3A** Correlations among traits Whole, Yield, and DG (Desired Gains index, year 2020) for weights $\mathbf{d}'=\left[\begin{matrix} -1 & 1 \end{matrix}\right]$ (Fig. A) and $\mathbf{d}'=\left[\begin{matrix} -10 & 70 \end{matrix}\right]$ (Fig. B); correlations among traits Whole, Ratoon, Yield, and DG (year 2021) for weights **d** = [-1  1  1] (Fig. C) and **d** = [-10  10  70] (Fig. D).

**Supplementary Material B**

*The expected genetic gain per trait theory*

Kempthorne and Nordskog (1959) defined the expected genetic gain per trait assuming that $I$and $\mathbf{g}$ have joint multivariate normal distribution, in a similar manner as Smith (1936) did it when developed the index theory. Under such assumption, the expected genetic gain per trait is

$\mathbf{E}=p^{-1}E\left[E(\mathbf{c}_{i}|\mathbf{b}'\mathbf{y}_{i})\right]=\frac{\mathbf{Cb}}{\sqrt{\mathbf{b}'\mathbf{Pb}}}p^{-1}E\left[\frac{\mathbf{b}'(\mathbf{y}_{i}-\mathbf{\mu})}{\sqrt{\mathbf{b}'\mathbf{Pb}}}\right]=\frac{\mathbf{Cb}}{\sqrt{\mathbf{b}'\mathbf{Pb}}}k$ , (S1)

where, according to Kempthorne and Nordskog (1959), $\mathbf{b}=\mathbf{P}^{-1}\mathbf{Cw}$ is the Smith index vector of coefficients and $p$ is the proportion of the population of animal or plant lines that will be selected. When $\mathbf{C}$ and $\mathbf{P}$ are known, $\mathbf{E}$is optimum (Cerón-Rojas and Crossa 2022). Equation (S1) is a vector $t\times1$ ($t=$ number of traits) of expected genetic gain values, not a scalar, and indicates that the problem of predicting the mean of $\mathbf{c}_{i}$ is simply estimating its conditional mean. Moreover, $\mathrm{cov}(\mathbf{c}_{i},\mathbf{y}'_{i}\mathbf{b})/\mathrm{var}(\mathbf{b}'\mathbf{y}_{i})$ is the regression of $\mathbf{c}_{i}$ on Smith index, and, in addition,

$$k=p^{-1}E\left[\frac{\mathbf{b}'(\mathbf{y}_{i}-\mathbf{\mu})}{\sqrt{\mathbf{b}'\mathbf{Pb}}}\right]=\frac{\exp\{-0.5\tau_{i}^{2}\}}{p\sqrt{2\pi}}=\frac{z(\tau_{i}^{2})}{p}$$

is the selection intensity, $\tau_{i}=\frac{\mathbf{b}'(\mathbf{y}_{i}-\mathbf{\mu})}{\sqrt{\mathbf{b}'\mathbf{Pb}}}$; $\sqrt{\mathbf{b}'\mathbf{Pb}}$ is the standard deviation of the variance of $I_{i}=\mathbf{b}'\mathbf{y}_{i}$, $\mathbf{\mu}$ was defined earlier, and $z(\tau_{i}^{2})=\frac{\exp\{-0.5\tau_{i}^{2}\}}{\sqrt{2\pi}}$ is the height of the ordinate of the normal curve at the lowest value of $\tau_{i}^{2}$ retained.

Kempthorne and Nordskog (1959) defined $\mathbf{E}$to control the average of the trait of interest imposing null constrains on the covariance between $\mathbf{c}_{i}$and $I_{i}=\mathbf{b}'\mathbf{y}_{i}$ for some traits and developed a Smith (1936) restricted selection index, or a restricted linear phenotypic selection index, denoted as RLPSI by Cerón-Rojas and Crossa (2018, Chapter 3). The RLPSI allows imposing restrictions equal to zero on the expected genetic gains of some traits, while other traits increase (or decrease) their expected genetic gains without imposing any restrictions. The RLPSI solves the Smith (1936) equations subject to the condition that the covariance between the index and some linear functions of the genotypes involved be zero, thus preventing selection on the RLPSI from causing any genetic change in some trait expected genetic gains. Cerón-Rojas *et al*. (2016b) showed that the RLPSI vector of coefficients can be written as

$$\mathbf{b}_{R}=\mathbf{Kb}$$

where $\mathbf{b}=\mathbf{P}^{-1}\mathbf{Cw}$and $\mathbf{K}$ is an idempotent matrix ($\mathbf{K}=\mathbf{K}^{2}$) which projects $\mathbf{b}$ into a space smaller than the original space of $\mathbf{b}$ because the restrictions imposed on the expected genetic gains per trait are equal to zero. The reduction of the space into which matrix $\mathbf{K}$ projects $\mathbf{b}$ will be equal to the number of null restrictions imposed by the breeder on the expected genetic gain per trait.
